# Outcome strategies for clinical trials in Neuropaediatric rare diseases

**DOI:** 10.1016/j.nsa.2026.107021

**Published:** 2026-07-10

**Authors:** Maria Teresa Acosta, José Ángel Aibar, Celso Arango, Estibaliz Arce Cirauqui, Cristina Baeza, Elizabeth Berry-Kravis, Kim I. Bishop, Joan Busner, Chere Chapman, Boris Chaumette, Inés del Cerro, John Carlos Diaz, Georg Dorffner, Chris J. Edgar, Simona Giorgi, Sabine M. Hölter, Sarah Kittel-Schneider, Robert D. Latzman, Carmen Moreno, Stefano Pallanti, Carlotta Colzi, Carme Plasencia, Franziska Radtke, Kenneth Rockwood, Antonella Santuccione Chadha, Gunes Sevinc, Manpreet K. Singh, Suzanne H. Smith, P.K. Tandon, Daniella Tinoco, Eleonora Broggi, Sara Fontecha Morgan, Igor Magaraggia, Silvia Zaragoza Domingo

**Affiliations:** a NHGRI, Undiagnosed Disease Programme, National Human Genome Research Institute, NIH, Bethesda, USA; b Dravet Syndrome Foundation Spain, Madrid, Spain; c Department of Child and Adolescent Psychiatry, Hospital Universitario La Paz, IdiPaz, School of Medicine, Universidad Autónoma de Madrid, CIBERSAM, Madrid, Spain; d Engrail Therapeutics Inc, San Diego, CA, USA; e CBA Educational Training and Health Consulting, Barcelona, Spain; f Departments of Pediatrics, Neurological Sciences, Anatomy and Cell Biology, Rush Medical Centre, Chicago, IL, USA; g F.A.S.T. Center for Translational Research, Rush University Medical Center, Chicago, IL, USA; h Global Pharma Consultancy, Muncy, PA, USA; i Signant Health, Blue Bell, PA, USA; j Department of Psychiatry, Virginia Commonwealth University School of Medicine, Richmond, VA, USA; k Ardea Outcomes, Halifax, Nova Scotia, Canada; l Institute of Psychiatry and Neuroscience of Paris (INSERM U1266), Institut Pasteur (CNRS UMR3571), GHU-Paris Psychiatrie et Neurosciences, Paris, France; m Catholic University of Murcia (UCAM), Murcia, Spain; n Geosera Ltd, Berwyn, IL, USA; o The Siesta Group, Vienna, Austria; p Center for Medical Data Science, Medical University of Vienna, Vienna, Austria; q PCOA Associates Ltd, UK; r Helmholtz Center Munich, German Research Centre for Environmental Health, Munich, Germany; s Department of Psychiatry and Neurobehavioural Science, University College Cork, Cork, Ireland; t Otsuka Pharmaceutical Development & Commercialization Inc, Princeton, NJ, USA; u Department of Child and Adolescent Psychiatry, Institute of Psychiatry and Mental Health, Hospital General Universitario Gregorio Maranon, School of Medicine, Universidad Complutense, IiSGM, CIBERSAM, ISCIII, Madrid, Spain; v Albert Einstein College of Medicine, USA; w Istituto di Neuroscienze, Firenze, Italy; x Applied Research using Omic Sciences S.L. (Aromics), Barcelona, Spain; y Department of Child and Adolescent Psychiatry, Psychosomatics and Psychotherapy, University Hospital of Würzburg, University of Würzburg, Würzburg, Germany; z Division of Geriatric Medicine, Dalhousie University, Halifax, Nova Scotia, Canada; aa Geriatric Medicine Research Unit, Nova Scotia Health Authority, Halifax, Nova Scotia, Canada; ab Women's Brain Foundation, Basel, Switzerland; ac Department of Psychiatry and Behavioural Sciences, University of California, Sacramento, CA, USA; ad Origins, part of the Resonant Group, London, UK; ae Ultragenyx Pharmaceutical Inc., Novato, CA, USA; af Independent Consultant, Barcelona, Spain; ag Department of Forensic and Neurodevelopmental Sciences, Institute of Psychiatry, Psychology and Neuroscience, King's College London, UK; ah Centre for Global Health, Trinity College Dublin, Ireland; ai Igor Magaraggia Medical Writing and Consulting, Verbania, Italy; aj Neuropsychological Research Organization S.L. (Neuropsynchro), Barcelona, Spain

**Keywords:** Neuropaediatric rare diseases, Clinical trials, Outcome measures, Clinical outcome assessments, Digital health technologies, Natural history data

## Abstract

Neuropaediatric Rare Diseases (NRDs) impose a profound and multidimensional burden on patients, families, and healthcare systems. Persistently low clinical trial success rates reflect an unmet methodological need as much as a therapeutic one. A fundamental bottleneck is the absence of fit-for-purpose clinical outcome assessments. In fact, instruments validated for non-rare or adult populations fail to capture clinically meaningful change in heterogeneous, small, and developmentally complex NRD populations. Building directly on a systematic catalogue of methodological challenges in NRD outcome research published by our group (Acosta et al., 2025), this paper presents a structured set of actionable outcome strategies proposed by a large multidisciplinary expert group convened under the auspices of the European College of Neuropsychopharmacology (ECNP) and the International Society for CNS Clinical Trials and Methodology (ISCTM). Using a structured, iterative expert-opinion approach, each identified challenge served as a prompt for developing one or more candidate strategies, each mapped one-to-one to its corresponding barrier. Strategies are presented across four thematic domains: (1) innovative methodologies to enhance ecological validity and reduce rater context effects; (2) novel or adapted outcomes and endpoints that preserve clinical meaningfulness under conditions of high heterogeneity and limited sample sizes; (3) the purposeful use of natural history resources; and (4) approaches to support comparability and synthesis across programmes. Additional considerations address caregiver expectancy bias and recruitment, stakeholder alignment, maturational confounding, and preclinical-clinical connectivity. Collectively, these strategies constitute a practical, challenge-mapped “living” toolbox for clinical scientists designing NRD trials. Each strategy is already in use or validated in analogous rare-disease contexts. Realising their potential at scale requires institutional programmes, pre-competitive co-validation platforms, systematic stakeholder co-design, and early engagement with regulatory agencies as scientific partners in endpoint development.

## Highlights

• Wearables and momentary assessment cut recall bias and add objective endpoints.
• Established methods can build novel endpoints for small, heterogeneous samples.
• Consensus core outcome sets in routine care strengthen small-sample trials.
• Structured mining of medical records yields trial-ready natural-history data.
• Natural history cohorts may serve as external controls in single-arm NRD trials.

## Graphical abstract

**Figure:**
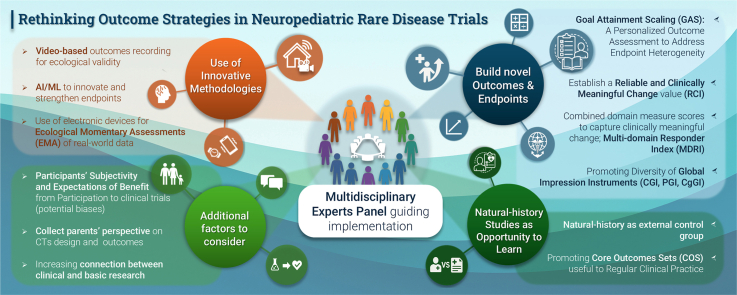

## 1 Introduction

Neuropaediatric Rare Diseases (NRDs) are defined as rare conditions that primarily manifest between birth and 18 years of age and affect the central or peripheral nervous system. A large proportion of NRDs are of genetic or genomic origin, and children with severe intellectual disabilities are significantly more likely to harbour an identifiable genetic aetiology than their neurotypical peers (Acosta, 2023; Gahl et al., 2016). Clinically, NRDs are characterized by early onset, frequently progressive or fatal trajectories, prolonged periods of functional dependence, and substantial reductions in quality of life across cognitive, motor, communicative, and behavioural domains (Hadders-Algra, 2021). Despite advances in molecular genetics, therapeutic science, and trial design, effective therapies remain unavailable for the vast majority of NRDs, and persistently low clinical trial success rates reflect an unmet methodological need as much as a therapeutic one (Kioutchoukova et al., 2023).

Among the most consequential of these methodological challenges is the selection, design, and validation of appropriate outcome measures. Any clinical outcome assessment (COA) instrument employed in a clinical trial must satisfy two fundamental requirements: it must capture change that is clinically meaningful to patients, caregivers, and clinicians, and it must demonstrate robust psychometric properties, including validity, reliability, and responsiveness to change (Esbensen and Schworer, 2022; Farmer et al., 2022). However, satisfying these requirements in NRDs is complicated by a constellation of barriers. In 2023, an international multidisciplinary expert group convened to examine outcome-related barriers across NRD clinical programmes, building on prior work on endpoint strategy, stakeholder engagement, and paediatric drug development (Acosta et al., 2025; Busner et al., 2021, 2023; Harvey et al., 2023; Pandina et al., 2023, 2024). This effort resulted in a catalogue of 28 distinct gaps in COA research, including frequent floor and ceiling effects in standardized instruments, inconsistent measurement practices across sites, limited regulatory precedent for innovative endpoints, and the risk of expectation-induced bias by caregivers and clinicians alike (Acosta et al., 2025) Additionally, the work highlighted the weak linkage between biological targets and outcome measurement, adoption of endpoints borrowed from non-rare-disease contexts, and the absence of tailored frameworks for decision-making under conditions of extreme sample scarcity (Table 1; for further details on the stakeholder landscape and COA selection strategies underpinning this work, see Supplementary Fig. 1).

**Table 1:** Key takeaways from a recent targeted literature review in Neuropaediatric Rare Disorders (Acosta et al., 2025).

Key takeaways
NRDs represent a field with high stakeholder interaction, in which patient advocacy plays a central driving role.
Innovative COA solutions can be transversal, applicable across different rare disease therapeutic areas that share similar research needs.
Pre-competitive efforts are relevant to accelerating COA innovation and tool development.
Sustainability of innovation through public or private investment is necessary to advance methodological solutions.
Summarizing and evaluating existing NRD resources helps identify synergies and minimize duplication of effort when defining an action roadmap.
Given the limited number of patients, future solutions are needed to expedite the collaborative validation and qualification of novel COA instruments.

**Abbreviations:** COA, Clinical Outcome Assessment; NRD, Neuropaediatric Rare Disease.

The present work outlines actionable outcome strategies identified through group discussions and constitutes a direct follow-up to the prior (Acosta et al., 2025). These strategies were identified by a multidisciplinary expert group that convened under the joint auspices of the European College of Neuropsychopharmacology (ECNP) Network on Clinical Outcomes Research in Early-Phase Trials and the Orphan Diseases Working Group of the International Society for CNS Clinical Trials and Methodology (ISCTM), comprising academic clinicians, pharmaceutical and biotechnology industry professionals, contract research organization staff, patient advocacy representatives, and outcomes and regulatory science specialists. The methodological approach was structured, iterative expert opinion rather than a systematic review: each challenge catalogued in the companion publication served as a prompt for the group to identify and develop one or more candidate outcome strategies, each mapped one-to-one to its corresponding barrier (for full deliberation notes and candidate strategy rationales, see Supplementary Table 1; (Acosta et al., 2025).

Strategies are grouped under four thematic domains: (1) innovative methodologies technology-based to enhance ecological validity and reduce rater context effects; (2) novel or adapted outcomes and endpoints that preserve clinical meaningfulness under conditions of high heterogeneity and limited sample sizes; (3) the purposeful use of natural history resources; and (4) approaches to support comparability and synthesis across programmes adopting core outcomes set methodologies. Additional considerations address caregiver expectancy bias, trial recruitment, stakeholder alignment, maturational confounding, and preclinical-clinical connectivity. For each strategy, a brief overview, illustrative examples, and expected regulatory considerations are provided. Table 2 summarizes the main strategies, including both adaptations of existing endpoints and proposals for novel ones.

**Table 2:** Overview of novel and adapted outcome measurement strategies and endpoints for neuropaediatric rare disease clinical trials.

When	Proposed Outcome Strategy Name		Background	Considerations for Implementation
**A global measure is needed** Used as Anchors to show the meaningfulness of results: CGI is needed to validate ClinROs PGIs help interpret the meaningful benefit	**CGIs** **PGI/CgGI**	**Global Impression Types** (Guy, 1976; Busner and Targum, 2007)	Based on CGI-S/I/C from NIMH	• Customizable to condition • Global and dimensional • Grid description anchors • Vignettes with case examples • In some trials, a blinded rater is included.
**Complex multidimensional conditions evolving differently** Interest in capturing any change on any key dimension to document clinical benefit. Individual changes can be diluted. Dimensions can be weighted according to disease or to the MoA target	**MDRI**	**Multi-domain Responder Index** (Tandon and Kakkis, 2021)	Condition with different dimensions and heterogeneity in clinical evolution.	• Grid of dimensions and descriptions. • Set thresholds for meaningful change • Grid for calibration ratings with vignettes.
**Heterogeneous baseline and course severity** When baseline severity is heterogeneous, personalising outcomes makes it difficult to measure progress with a single method.	**GAS**	**Goal Attainment Scaling** (Kiresuk and Sherman, 1968; Kiresuk et al., 2014)	Each patient sets personalized goals.	• Interviews to set goals. • Training on how to evaluate evolution.
**True individual change over time** • Analysis of change in longitudinal data. Several factors can influence repeated measures; a reliable change in a group excludes measurement error. • Need to define a change that is meaningful based on the true group performance longitudinal data.	**RCI**	**Reliable Change Index**^a^ (Heilbronner et al., 2010)	Intention to compensate for the group average for idiosyncratic individual or practice-effect variation.	• Substitute the usual calculation of the change average with an RCI. • Use this RCI for all change calculations.
**Summary of change at group level over time** • When normative data does not exist or is impossible to obtain. • Normative population is not a good reference for patients with a specific condition.	**GSV**	**Growth Scale Value**^b^ (Edwards, 2009; Farmer et al., 2020; Kean et al., 2018)	Use robust methods such as IRT to better characterize performance progression rather than relying on normative data.	• Calculation based on large data sets of follow-up of the target population. • It can be calculated in all performance tests, although is an ad hoc complementary information

Abbreviations: CgGI, Caregiver Global Impression; CGI, Clinical Global Impression; CGI-I/C, CGI of Improvement/Change; CGI-S, CGI of Severity; COA, Clinical Outcome Assessment; GSV, Growth Scale Value; IRT, Item Response Theory; MoA, Mechanism of Action; NIMH, National Institute of Mental Health; MCID, Minimally Clinically Important Difference; MID, Minimally Important Difference; NRD, Neuropaediatric Rare Disease; PGI, Patient Global Impression; RCI, Reliable Change Index; SEM, Standard Error of the Mean.

a Typically calculated as the difference between post and pre score divided by the standard error of the difference; change is considered “reliable” if the absolute RCI exceeds a z-criterion such as 1.96 (≈95% confidence), or equivalently if the raw change exceeds a “coefficient of repeatability” threshold.

b Test-specific scores are placed on an equal-interval developmental scale so that differences in score units have the same meaning across the continuum, allowing direct comparison of growth over time. In summary, Growth Scale Value summarizes change, while RCI tests whether an individual change is reliable.

## 2 Use of innovative methodologies

Conventional clinical assessments in NRDs are limited by the clinical environment, where patient unfamiliarity, white-coat effects, and brief visits can alter behaviour and bias measures of how patients feel and function (Pioli et al., 2018). Given the small populations and high phenotypic variability that characterize NRDs, assessment methods must offer exceptional sensitivity, flexibility, and ecological validity. This section introduces complementary approaches to address these challenges.

### 2.1 Video-based Outcomes Recording for Ecological Validity

Clinical assessments conducted in hospitals or research settings often fail to capture how patients truly function in daily life, whereas parents routinely observe a broader and more natural range of behaviours at home (Pioli et al., 2018; Bennetts et al., 2016). Standardized home-based video recordings, in which caregivers follow scripted instructions to film predefined daily tasks aligned with the patient's baseline abilities, help bridge this gap by enabling trained researchers to evaluate changes in functioning over time. These tasks can be embedded in everyday routines—such as dressing, toothbrushing, feeding, or mobility—and scripts ensure consistency across multiple recordings, whether families submit several attempts or contribute repeated videos for larger datasets. Scoring can be performed using tools like the open-source Behavioural Observation Research Interactive Software (BORIS), which supports structured coding, event logging, and inter-rater reliability assessment (Friard and Gamba, 2016).

This approach has proven feasible in multiple clinical and regulatory contexts. For example, the U.S. Food and Drug Administration (FDA) accepted video-based mobility testing to assess real-world functional vision in the Luxturna programme (voretigene neparvovec-rzyl) for retinal dystrophy (Chung et al., 2018; Russell et al., 2017). Similarly, the Scale for the Assessment of Ataxia SARA-home ataxia protocol enables patient-recorded tasks aligned with validated in-clinic scales (Davies et al., 2022). Finally, a video-based rating has been used to distinguish tics from stereotypies in severe autism spectrum disorder (Termine et al., 2021).

Regulatory bodies, including the FDA, the European Medicines Agency (EMA), and Health Technology Assessment (HTA) groups such as NICE,^1^ increasingly recognize video-derived, patient-centered data—nwhen supported by validation and inter-rater reliability—as valuable endpoints in rare disease trials (Davies et al., 2022; Jadoenathmisier et al., 2025; U.S. Food and Drug Administration). Key implementation challenges, such as variability in parental adherence to scripts, the risk of selective editing, and the complexity of harmonizing procedures across international sites, can be mitigated through precise scripting, centralized blinded scoring, predefined validation plans, and collaborative standardization with clinicians, caregivers, and regulatory stakeholders.

### 2.2 Artificial Intelligence (AI) and Machine Learning (ML) to strengthen NRD trial endpoints

Conventional trial endpoints in NRDs are often episodic, noisy, and poorly suited to detecting short-term biological change. AI/ML methods offer opportunities to develop endpoints with greater precision by identifying complex, multivariate, and potentially non-linear patterns in clinical data that outperform individual biomarkers or single-scale scores.

Three categories of AI/ML applications are relevant to NRD outcome research:

1. **Classical machine learning** includes methods such as random forests, support vector machines, and simple neural networks. When applied to standard clinical features (e.g., physiological measurements), these methods can identify “composite biomarkers” with higher predictive value than any single variable (Agibetov et al., 2020). However, in practice, these methods rarely uncover true non-linearity, and linear multivariable models often perform similarly (Christodoulou et al., 2019).
2. **Deep learning** applied to structured raw data (e.g., neuroimages, video recordings) can detect clinically meaningful patterns in high-dimensional data. For example, deep neural networks trained on polysomnographic recordings have achieved near-expert diagnostic performance for narcolepsy (Stephansen et al., 2018), demonstrating the potential of this approach in NRDs and other clinical conditions.
3. **Large language models (LLMs)** excel at extracting and synthesizing information from text and multi-modal sources and can support knowledge interaction and clinical documentation. However, they are currently the least applicable to developing quantitative endpoints from trial data.

For classical ML and deep learning approaches, large, well-annotated patient cohorts are essential for developing and validating AI-derived endpoints. Because NRDs are low-prevalence, generating sufficiently large datasets is challenging. International multi-site registries, combined with the home-based data collection described in Sections 1.1 and 1.3, represent the most feasible path to achieving adequate sample sizes. AI/ML approaches have relevance for personalized medicine. Even modest population-level data sets can capture variability patterns that support individual-level modelling (Randhawa and Sigalov, 2025), which is particularly important for NRD trials that may include only a handful of participants or even a single patient. Finally, using AI-based analytical frameworks in regulatory settings will require expanded FDA and EMA guidance tailored to the specific challenges of NRDs and AI-based endpoints (EMA and FDA set common).

### 2.3 Ecological momentary assessment using Electronic Devices

Clinic-based evaluations offer only brief, context-dependent snapshots of patient functioning. The repeated, real-time measurement of experience, behaviour, and function in natural environments, now termed ecological momentary assessment, allows dense longitudinal data collection outside the clinic.

Passive remote monitoring has long been used in neurodevelopmental conditions. For example, actigraphy to measure hyperactivity in attention-deficit/hyperactivity disorder has been documented since 1992 (HALPERIN et al., 1992), and remains common in research across conditions (Ziegler et al., 2025).

Modern smartphones, smartwatches, and wearable sensors now enable continuous collection of activity and mobility metrics, autonomic and cardiac signals, sleep parameters, movement abnormalities such as tremor, and safety indicators such as fall detection. Large virtual cohort efforts, such as the Apple Health Study, demonstrate the feasibility of large-scale longitudinal monitoring using integrated data from smartphones, smartwatches, and headphones (Apple).

Ecological momentary assessments of parents and caregivers further capture context-rich observations tied to daily routines, including behaviour, participation, mood, and communication, supported by platforms with scheduled prompts and automated data transfer. This methodology has already been incorporated into clinical trials; for example, the Q1.6 app used in clinical trials (e.g., NCT03559192) alerts investigators to near-real-time changes in depressive symptoms, and other trial-specific tools follow similar models.

Effective implementation of ecological momentary assessment requires that endpoints reflect patient- and caregiver-identified concepts of meaningful health, and that feasibility and user acceptance are confirmed in the target population before pivotal use. An international consensus initiative recently developed a quality framework for item design (Eisele et al., 2025), offering standards suitable for NRDs. Pre-competitive multi-site collaborations provide an efficient path for developing and validating standardized protocols across rare disease subgroups that share methodological needs.

## 3 Building novel outcomes and endpoints

Population-level endpoints designed for common use often fail in NRD trials due to high phenotypic heterogeneity, small sample sizes, and frequent floor effects of standardized instruments. As a result, genuine treatment effects may go undetected, and clinically important changes for individual patients can be masked by group-level averaging (Acosta et al., 2025). This section introduces five strategies designed to capture meaningful change under these conditions, each addressing a specific yet often overlapping methodological barrier.

### 3.1 Goal Attainment Scaling for Personalized Endpoints

**Fig. 1:**
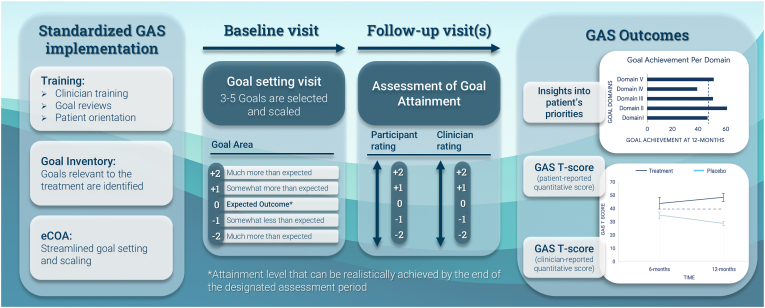
Standardized implementation of Goal Attainment Scaling (GAS) through training, use of a goal inventory and an electronic COA platform. GAS outcomes capture the treatment effects that are most meaningful to patients. At the same time, they provide a structured framework for evaluating intervention effectiveness in terms of clinically meaningful change, as reported by both patients and clinicians, derived through dialogue.

Standardized clinician-reported and observer-reported (ClinROs and ObsROs) instruments often lack sensitivity in NRDs because they include items that are irrelevant or redundant for many patients (Benjamin et al., 2017; Silva et al., 2024). Goal attainment scaling (GAS) addresses this limitation by tailoring outcomes to each individual's symptom profile and functional baseline (Fig. 1).

In GAS, patients or caregivers collaborate with a clinician to define three to five personalized, treatment-relevant goals that reflect the symptoms, functions, or daily challenges most meaningful to them (Kiresuk and Sherman, 1968; Kiresuk et al., 2014). The resulting standardized summary score, which adjusts for the number of goals and any inter-goal correlations, supports both within-patient and between-patient comparisons, enabling group-level analysis while retaining individual relevance (Gaasterland et al., 2019; Cheema et al., 2024).

Importantly, GAS can be configured to capture the stabilisation of function as a positive endpoint in progressive or neurodegenerative conditions, offering the flexibility that most standardized COAs lack.

Implementation of GAS in NRD begins with developing a condition-specific goal inventory (Goldstine et al., 2021; Staunton et al., 2025) and confirming feasibility before use in pivotal studies (Sevinc et al., 2025). Standardized procedures, including clinician training, patient and caregiver orientation, and ongoing data monitoring, are essential to ensure data quality and interpretability. Semi-structured goal inventories help align selected goals with treatment's mechanism of action, as was highlighted in the FDA Patient-Focused Drug Development guidance (Sevinc et al., 2024; Roberts et al., 2022; U.S. Food & Drug Administration (FDA)).

In single-arm designs, which are frequently necessary in NRDs, GAS implementation must also address expectancy bias. Strategies such as non-treatment-related control goals, blinded goal raters, and delayed assessment windows can help mitigate this risk (Murray et al., 2023).

Real-world feasibility has been demonstrated in a feasibility study for SCN2A-associated developmental and epileptic encephalopathy (Sevinc et al., 2025), and also in clinical trials as in refractory epilepsy (CBD-uN1que) (NCT07668856). While rigorous methodological controls remain essential for regulatory use, GAS's ability to measure outcomes that matter most to individual patients is a key strength supporting its broader adoption in NRD clinical development.

### 3.2 Clinical Global Impression instruments adapted for NRDs

The Clinical Global Impression (CGI) scale, originally developed for psychiatric conditions, provides a brief holistic assessment of overall disease severity (CGI-S) and treatment-related changes (CGI-I/C) (Guy, 1976; Busner and Targum, 2007). Its face validity, broad applicability, and interpretability among stakeholders make it valuable for phenotypically heterogeneous NRDs, where narrow, domain-specific measures may miss meaningful change. Patient- and caregiver-reported analogues (PGI-S/I/C and CgGI-S/I/C) extend this framework to capture family perspectives.

However, standard CGI Instruments require careful adaptation for NRD. Without condition-specific behavioural anchors and structured rater training, CGIs are vulnerable to expectation bias, construct drift, and inconsistent scoring across sites. Reliable implementation, therefore, depends on pre-specified developmental and disease-specific anchors, calibration vignettes and rater-qualification procedures to ensure consistent interpretation, particularly in progressive phenotypes where expected developmental decline may confound ratings (Table 3).

**Table 3:** Tips for customizing Clinical Global Impression scales for Neuropaediatric Rare Disease clinical trials.

Tips for Customizing Clinical Global Impression (CGIs) Scales
1. Tailoring the description of the anchors to the condition and to the developmental stage, if needed, helps reduce construct drift (e.g., ensuring decrements in expected abilities, such as ambulation in progressive phenotypes, do not disproportionately bias the global score).
2. Multidimensional global instruments can combine a single global item with a small set of disease-salient domains (e.g., communication, motor, behaviour), yielding a transparent profile while preserving a global summary for endpoints or anchors. These disease-specific variants can be shared across centres for iterative validation.
3. Define disease-specific anchors linked to concrete behaviours and caregiver priorities.
4. The lower level of severity needs to be named level 0, corresponding to “Normal, Not ill”, instead of naming it level 1, which may lead to confusion during data analysis.
5. Consider a multidimensional global with a single summary plus a few core dimensions, importantly keeping the “global” aspect of the scale.
6. Pre-define analysis rules (e.g., handling of expected developmental decline).

NRD-adapted CGI frameworks may combine a single global item with a small set of disease-salient domains (e.g., communication, motor function, behaviour) to preserve the holistic perspective while providing transparent domain-level insights. Established interpretive conventions for CGI-I scores (e.g., scores of 1–3 indicating clinical benefit) support regulatory review, and prior disease-specific adaptations—such as the Rett syndrome CGI (Neul et al., 2015)—provide successful precedents.

Retrospective CGI scoring (R-CGI) provides an alternative when prospective global assessments are not embedded in routine care. A recent application in GM1 gangliosidosis demonstrated its feasibility for quantifying historical control data under structured validation procedures (Lewis et al., 2025). With disciplined adaptation and training, CGIs remain a versatile, regulator-recognized tool for capturing clinically meaningful global change in NRD trials.

### 3.3 Building combined scores from multi-domain endpoints

NRDs are multisystem conditions affecting cognitive, motor, communicative, behavioural, and functional domains. Single-domain endpoints are often inadequate because they require selecting one domain a priori, exclude patients who lack that specific deficit and fail to capture aggregate treatment benefit across a broader clinical profile (Tandon and Kakkis, 2021).

The Multi-Domain Responder Index (MDRI) addresses this limitation by quantifying clinically meaningful change across multiple prespecified domains.

**Fig. 2:**
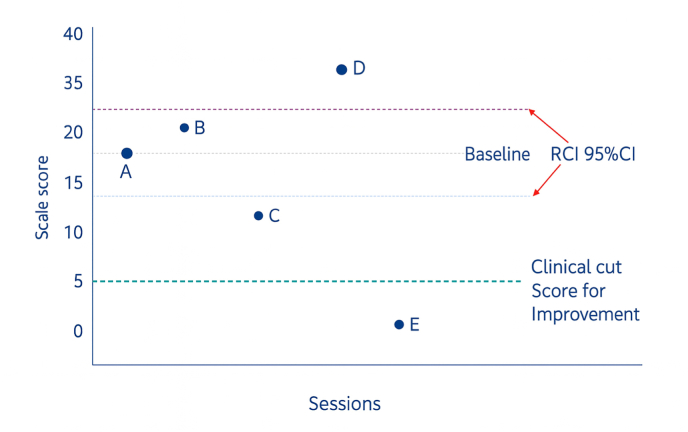
Illustrative Example of MDRI result (adapted with permission from (Tandon and Kakkis, 2021)). Abbreviations: Multi-Domain Responder Index, MDRI.

For each patient, the MDRI determines whether a change in each domain exceeds a predefined minimum score difference (MSD). Each domain is scored as a binary responder/non-responder, and domain-level responses are summed to generate a net response score that serves as the efficacy variable (Tandon and Kakkis, 2021). This approach accommodates heterogeneous phenotypes, broadens eligibility criteria, and preserves statistical power in small samples. An example of implementing the MDRI methodology is shown in Fig. 2.

Two regulatory precedents support MDRI feasibility. In Mucopolysaccharidosis (MPS) I and VII, MDRI-based analyses captured multi-domain clinical benefit and contributed to labelling decisions (Wraith et al., 2004). MDRI has also been prospectively integrated as a key secondary endpoint in a Phase 3 trial in Angelman syndrome (NCT06617429).

The principal implementation challenge is determining MSD thresholds for each domain. Natural history data, published clinical benchmarks, and sensitivity analyses varying thresholds and domain weights provide the most defensible foundation. With adequate calibration, the MDRI becomes a valuable approach for capturing global treatment benefit in complex NRD presentations.

### 3.4 Establishing Reliable and Clinically Meaningful Change Values

**Fig. 3:**
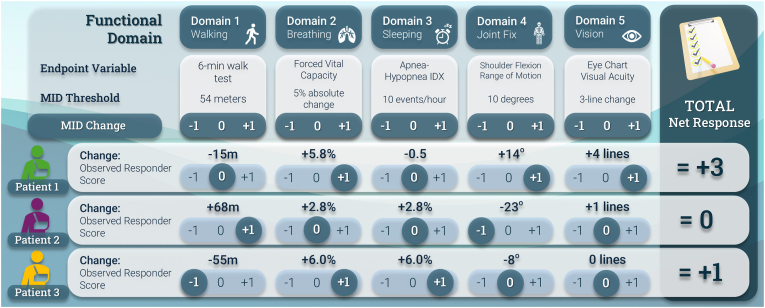
Illustrative fictive example of changes in the Reliable Change Index (RCI) for an individual patient over different assessment sessions (adapted with permission from (Morley and Dowzer, 2014)). Abbreviations: Confidence Interval, CI; Reliable Change Index, RCI.

NRD trials often rely on small samples and include patients who score at or near the floor of standardized measures, making group-mean comparisons an insensitive strategy for detecting treatment effects (Acosta et al., 2025). The reliable change index (RCI) offers a complementary approach by determining whether an individual patient's change exceeds what would be expected from measurement error, practice effects or natural variability (Fig. 3 (Heilbronner et al., 2010);). This individual-level focus is particularly valuable in heterogeneous and low-incidence NRD populations.

RCI classification is typically based on whether the magnitude of change between two assessments exceeds the standard error of the difference, with RCI ≥1.96 indicating a 95% confidence that the change is reliable. Modified RCI methods can adjust for practice effects (Chelune et al., 1993) or accommodate multi-timepoint designs using regression-based approaches (McSweeny et al., 1993; Combs et al., 2021).

Reliable change alone does not guarantee clinical meaningfulness, but it provides a principled foundation for identifying potential responders. In NRD research, RCI has been successfully applied, for example, in a Phase I trial of lovastatin for neurocognitive deficits in neurofibromatosis type 1, where individual-level analyses revealed improvements not apparent in group means (Acosta et al., 2011; Hawksworth et al., 2024). For n-of-1 and single-arm designs increasingly used in NRDs, combining RCI with slope-based change metrics offers a rigorous alternative to between-group comparisons (Hawksworth et al., 2024).

Developing RCI benchmarks for commonly used neurocognitive and behavioural instruments in NRD trials represents a low-resource, high-value contribution. These metrics can be incorporated prospectively as analytic decision rules or applied post-hoc as exploratory endpoints to support a more nuanced interpretation of treatment effects.

### 3.5 Growth Scale Value (GSVs) for floor-effect-resistant measurement

Children with NRDs often score at or near the floor of standardized developmental and neuropsychological assessments, masking genuine improvements in skill acquisition. Derived from item response theory (IRT) and Rasch modelling, Growth Scale Values (GSVs) address this limitation by estimating a patient's absolute ability on an interval-level scale that is independent of normative reference groups (Edwards, 2009; Farmer et al., 2020; Kean et al., 2018).

GSVs place item difficulty and person ability on a shared latent continuum calibrated from large datasets. A one-unit GSV difference corresponds to the same magnitude of change in ability across the full range of functioning, enabling precise longitudinal tracking even when standard scores remain at the normative floor. This makes GSVs particularly useful for n-of-1 and single-arm NRD trials, where slope-based change analysis provides a robust alternative to group means (Hawksworth et al., 2024).

Active NRD applications include the use of Vineland-3 and Bayley GSVs in Fragile X syndrome (NCT05030129), autism (NCT05067582) and antisense oligonucleotide n-of-1 trials. A key next step is establishing clinically meaningful thresholds—how many GSV units correspond to a change that families and clinicians recognize as beneficial. Anchor-based, distribution-based, and qualitative methods (e.g., exit interviews) are appropriate strategies for defining these thresholds (Farmer et al., 2020).

## 4 Natural history studies as an opportunity to learn

For most NRDs, no approved therapy exists, and a conventional randomised placebo-controlled trial is often neither feasible nor ethically appropriate. In this context, natural history studies (NHS; longitudinal observation of the untreated disease course) serve not only as foundational infrastructure but also as the primary comparative benchmark for assessing therapeutic effects (Liu et al., 2022). Two complementary approaches are especially relevant: using prospective NHS cohorts as external control arms and extracting structured evidence from existing medical records.

### 4.1 Natural history data as external controls

NHS cohorts can function as an external control arm against which outcomes in a treated cohort are compared. Outcomes substantially better than expected based on the natural disease trajectory provide prima facie evidence of therapeutic benefit (Liu et al., 2022). Regulatory guidance from the FDA and EMA now acknowledges the role of both registries and NHS data in supporting decision-making for rare diseases (U.S. Food and Drug Administration, 2023; European Medicines Agency, 2021).

Recent approvals illustrate this trend: among non-oncology single-arm trial approvals between 2019 and 2022, natural history cohorts were the most frequent comparator, used in 45% of FDA and 47% of EMA decisions (Subramaniam et al., 2024). Programmes in Duchenne muscular dystrophy, spinal muscular atrophy, and Huntington's disease further demonstrate how NHSs can inform endpoint selection, phenotype evolution and eligibility criteria when designed with trial readiness in mind (Liu et al., 2022).

Despite their promise, external control arms designs present methodological challenges. Differences in patient selection, observation windows, instrument versions, and rater training introduce systematic bias, and the absence of randomisation increases the reliance on analytical assumptions. These risks can be mitigated through approaches such as propensity score matching, covariate adjustment and sensitivity analysis, but their validity depends on the quality and granularity of the underlying data. Therefore, NHSs should be prospectively designated using the same instruments, timepoints and outcome frameworks that future interventional trials are likely to require. For conditions in which genetic diagnosis precedes symptoms, enrolling patients at the time of molecular diagnosis enables the development of pre-symptomatic trajectories that further guide early-stage endpoint selection.

### 4.2 Extracting value from existing medical records

Clinical records accumulated in routine care offer an often-untapped source of longitudinal natural history data, frequently spanning years or decades of follow-up that no prospective study could feasibly replicate. Their primary limitations, including heterogeneous structure, variable completeness, and inconsistent linkage to standard outcome frameworks, are methodological rather than conceptual.

Nonetheless, structured retrospective extraction can transform these data into trial-relevant evidence. For example, a retrospective CGI scoring protocol (R-CGI-S/C) was successfully applied to a decade of NIH medical records in GM1 gangliosidosis type II, producing quantifiable trajectories that directly informed early-phase therapeutic development (Lewis et al., 2025; D'Souza et al., 2024).

Integrating such retrospective methods with prospectively designed COS-aligned data collection would produce directly comparable pre- and post-treatment windows, strengthening the natural history evidence base for future NRD programmes. In this regard, AI-assisted natural language processing offers a scalable complement to manual review, particularly for large multi-site datasets (Javed and Islam, 2025).

## 5 Urgent need for core outcome sets and clinical outcome assessments

The strategies described in Sections 1, 2, 3 are only effective if the outcome measures they rely on are validated and fit for the specific NRD context of use. Current practice, however, often depends on instruments borrowed from non-rare or adult populations, many of which lack the measurement properties required for NRD trials. A structural response to this limitation is the development of Core Outcome Sets (COS): consensus-based, minimum sets of outcomes that should be measured and reported in all trials for a given condition. COS reduce heterogeneity across studies, facilitates evidence synthesis, and ensures that research priorities reflect outcomes most meaningful to patients, caregivers, and clinicians.

### 5.1 Core outcome set initiatives in NRDs

Established frameworks already provide structured pathways for COS development that can be applied directly to NRDs. These include the COMET (Core Outcome Measures in Effectiveness Trials) Handbook (Williamson et al., 2017) and the ECNP/ISCTM 7-step COA development process (Zaragoza Domingo et al., 2024), which outlines systematic identification of candidate outcomes, multi-stakeholder engagement, consensus prioritisation, and prospective validation. Recent initiatives, such as the 2023 international Rett syndrome workshop, demonstrate how rigorous, condition-specific COA development pipelines can be built with explicit applicability to clinical trial settings (Downs et al., 2024).

COS development also benefits from a transdiagnostic approach. In fact, many NRDs share core domains of impairment, such as adaptive functioning, communication, motor skills, behavioural regulation, and caregiver burden. A modular COS structure, with a shared “backbone” set of outcomes plus condition-specific extensions, would reduce duplication across disease programmes, enable pooled validation datasets, and support regulatory harmonisation through a common conceptual framework. The RARE-X platform, a patient-driven initiative that enables longitudinal symptom and patient-reported data collection across rare diseases, provides an example of infrastructure aligned with this modular model and designed to address sample-size and data-access barriers (Vogel-Farley et al., 2025).

NRD clinical communities, such as in Phelan-McDermid syndrome, neurofibromatosis, and Dravet syndrome, are already showing informal convergence on common instruments and domains, indicating readiness for more formal COS efforts. For regulatory and HTA acceptance, COS must include clear context-of-use specifications, evidence of measurement properties in the target population, and interpretable thresholds for clinically meaningful change. The ECNP/ISCTM 7-step guide provides a robust starting point for structuring COS development in both clinical practice and natural history cohort design (Zaragoza Domingo et al., 2024).

## 6 Other factors influencing outcome research for NRDs

Beyond the technical dimensions of COA selection, several additional factors shape whether outcome strategies succeed in practice. These include caregiver expectancy bias, the challenges of recruiting and retaining families, the frequent misalignment between caregiver-valued outcomes and clinical endpoints, developmental confounding, and the weak linkage between preclinical and clinical research.

### 6.1 Managing caregiver expectations and supporting trial participation

Caregiver expectations can directly shape outcome measurement. Placebo arms in Fragile X syndrome trials showed significant gains on caregiver-rated endpoints but none on performance-based measures (Luu et al., 2020), and a Phelan-McDermid syndrome trial explicitly cited expectancy bias from parent-reported outcomes as a limitation (Kolevzon et al., 2022). This mirrors the broader informant-discrepancy literature, where caregiver–objective divergence is systematic rather than noise (De Los Reyes et al., 2013).

A practical mitigation strategy is to capture caregiver expectancy directly and adjust for it. However, no validated "Caregiver Expectancy Scale" exists as of today. A more actionable option is a caregiver-facing adaptation of the Patient Global Impression of Benefit–Risk (PGI-BR (Eek et al., 2021) into a Caregiver Global Impression of Benefit–Risk (CgGI-BR) which can be administered at baseline and entered as a covariate to adjust the estimated treatment effect for baseline expectancy.

### 6.2 Recruitment challenges

Recruitment in NRD trials requires tailored approaches due to small populations, high caregiver burden, and logistical barriers. Qualitative studies in Duchenne muscular dystrophy highlight the value of a dedicated coordinator who provides clear communication and continuity throughout the trial, enhancing participation and retention (Bendixen et al., 2016). Co-developed study materials, flexible visit schedules, telehealth assessments, home-based data collection, and wearable devices can significantly reduce burden and improve feasibility for families (Reaney et al., 2020).

### 6.3 Aligning caregiver and clinician outcome priorities

Clinicians and caregivers often prioritise different outcomes. Clinicians focus on signs, symptoms, and physiological disease activity, while caregivers emphasise meaningful daily functioning such as communication, participation, and independence (Vanderloo et al., 2021; De Wit et al., 2014).

In several NRDs, such as developmental and epileptic encephalopathies and Dravet syndrome, caregivers identify behavioural, cognitive, and social outcomes as more important than seizure frequency or other traditional clinical markers (Marchese et al., 2021; Darra et al., 2019; Dlugos et al., 2025).

Structural involvement of caregivers is therefore essential. Effective approaches include caregiver advisory boards, the inclusion of expert caregivers in protocol development, the validation of caregiver-reported measures through pre-competitive platforms, and the systematic use of exit interviews (Table 4). A meta-analysis found that collaborative co-design—not merely consultation—improves recruitment, retention, and dissemination in rare-disease research (Forsythe et al., 2014).

**Table 4:** Tips for collecting patients' and caregivers’ perspectives when designing a clinical outcome strategy for neuropaediatric rare disease clinical trials.

Tips to Collect Patients' & Caregivers' Perspectives in Clinical Trials
1. Create caregiver advisory boards to co-design inclusion criteria, outcome measures, and visit schedules.
2. Include expert caregivers as contributors in trial protocol design and IRB submissions.
3. Co-develop and promote the validation of disease-specific and caregiver-reported outcome measures.
4. Use multimedia tools to explain complex therapies and pilot them with families using available contexts (social, clinical, etc.).
5. Offer flexible participation options (e.g., telehealth, home visits, wearables) to increase diversity and inclusion.
6. Conduct structured exit interviews after clinical study completion and share post-trial summaries with patients and caregivers in accessible formats to increase the understanding of the benefits and safety of novel therapies.

### 6.4 A maturity index to disentangle treatment effects from natural development

Children with NRDs continue to develop biologically and neurodevelopmentally throughout a trial, making it difficult to distinguish treatment effects from expected maturation. Including a validated maturity index as a covariate can help partition observed changes into maturation-related and treatment-related components.

Bone age , determined through hand and wrist radiographs—now supported by AI-assisted methods to improve accuracy (Cavallo et al., 2021)—is a promising MI candidate. In settings where bone age progression correlates with functional gains in natural history data, adjusting for bone age change could refine efficacy estimates. Prospective validation in disease-specific cohorts is required to confirm whether BA or similar markers have sufficiently consistent relationships with target outcomes.

### 6.5 Strengthening the connection between preclinical and clinical research

Preclinical models—especially genetic mouse models—have been central to understanding NRD biology and generating early therapeutic hypotheses (Lange and Inal, 2023; Moro and Hanna-Rose, 2020). However, the translation gap remains wide for cognitive and behavioural NRDs, where higher-order functions depend on cortical architecture and environmental input that animal models cannot replicate (Acosta, 2013).

Emerging platforms help bridge this divide. INFRAFRONTIER provides systematic mouse phenotyping pipelines, offering structured access to cross-species data (da Silva-Buttkus et al., 2023; Ali Khan et al., 2023). In addition, GeneMatcher connects clinicians and researchers working on the same gene, facilitating rapid identification of phenotypic parallels (Sobreira et al., 2015; Calame et al., 2023).

These frameworks support targeted drug repurposing. A recent example is the clinically meaningful improvement in a child with Pitt–Hopkins syndrome following nicardipine, which was informed by mechanistic preclinical findings (Somorai et al., 2025). Coordinated, ethically governed data-sharing platforms linking clinical programmes with in vitro and in vivo models would accelerate such translational pathways and reduce reliance on chance collaborations.

## 7 Discussion

This paper presents a structured set of strategies for NRD clinical trials, developed through multidisciplinary expert collaboration and organised around the methodological challenges catalogued in our companion publication (Acosta et al., 2025). The proposed strategies span ecological data capture, construction of novel and hybrid endpoints, quantification of individual-level change, integration of natural history data, and additional considerations, including caregiver engagement, recruitment challenges, and translational gaps. Together, they form a coordinated response to well-defined barriers in NRD clinical research and provide practical guidance for designing more informative and patient-centered trials.

A noteworthy observation is the convergence between the strategies identified here and those proposed independently by other working groups. For example, Murray et al. (2023) emphasised the inadequacy of single-domain endpoints in heterogeneous rare-disease populations and the need for composite and personalized approaches—closely aligning with our recommendations. A recent systematic review of basket trials in rare diseases similarly highlighted harmonized endpoints, integration of real-world evidence, and composite outcome measures as tractable routes to increasing statistical power and regulatory acceptability in small-population trials (Khazen et al., 2025). That a separate group, working from different premises, reaches similar conclusions suggests that the field is beginning to coalesce around a shared understanding of its challenges and the methodological innovations needed to address them.

A central organising principle for applying this “toolbox” is the clinical trajectory of the target condition. NRDs can be broadly characterized as (1) neurodevelopmental and relatively stable, where the central deficit emerges early and remains mostly static (e.g., Fragile X syndrome), or (2) neurodevelopmental with neurodegenerative progression, where functional decline accumulates over time (e.g., GM1 gangliosidosis). This distinction has direct implications for endpoint strategy. Stable conditions require instruments sensitive to skill acquisition and functional gains against a developmental background. Progressive conditions, by contrast, require endpoints capable of capturing stabilisation or slowing of decline as meaningful clinical benefit, and natural history comparators that accurately reflect the expected deterioration. Sample size—often extremely small and sometimes limited to single-patient designs—determines the analytic framework (group-level versus within-person methods) but does not alter the relevance of these trajectory-based considerations.

Several limitations warrant acknowledgment. The strategies proposed here are based on structured expert opinion rather than prospective empirical validation and will require careful piloting and, when regulatory qualification is sought, formal validation studies. While broad in scope, the expert group represents a specific professional network and cannot capture all global perspectives. Moreover, the toolbox is not exhaustive; it reflects the challenges identified in a defined period of discussion and literature review, and new challenges will emerge as the science of NRDs advances. Supplementary Table 2 provides a working checklist of the challenges addressed and may serve as a planning resource for investigators as this evidence base continues to evolve.

The most urgent structural need is the creation of shared, pre-competitive platforms that enable researchers to co-validate outcome strategies and NRD-specific instruments. Many of the approaches described here are technically mature and feasible to implement today, but widespread adoption and regulatory confidence require larger validation datasets than any single programme can accumulate. Coordinated multi-site initiatives, spanning multiple NRDs and integrating natural history cohorts, represent the highest-leverage path forward. Equally important is engaging regulatory agencies early—as scientific partners rather than end-stage reviewers—to accelerate the iterative development and qualification of novel endpoints.

## 8 Conclusion

Clinical trials for NRDs face persistent and distinctive methodological challenges rooted in the inherent features of these conditions. Small and heterogeneous patient populations, variable developmental trajectories, and the limitations of standardized instruments demand more adaptive and sophisticated approaches to outcome measurement. The strategies presented in this paper are not theoretical proposals; each is already implemented in at least one NRD programme or validated in an analogous rare-disease context. Finally, it is worth noting that early engagement with regulatory agencies is a first step after deciding to use novel strategies.

The contribution of this expert group has been to synthesise these diverse methods into a coherent, challenge-mapped toolbox that investigators can selectively apply based on the specific features of the condition, the maturity of the therapeutic programme, and the regulatory context of use. The expectation is not that every strategy be used in every trial, but that they provide a structured, flexible foundation for designing fit-for-purpose endpoints.

Realising the full potential of these strategies requires three coordinated commitments from the field. First, trialists must pre-specify context-of-use and endpoint-validation plans early, rather than retrofitting outcome measures after data collection. Second, patient advocacy organizations, public funders, and industry partners must invest in pre-competitive platforms to co-validate NRD-specific instruments and share data across programmes. Third, regulatory agencies must be engaged early—as scientific partners rather than end-stage reviewers—to accelerate the development, refinement, and qualification of novel endpoints. Children with NRDs and their families cannot afford slow or fragmented progress. What is needed now is the coordinated, at-scale implementation of existing tools, considering all three recommendations, and, ideally, institutional programmes supporting all research needed to successfully promote the use of these methods in clinical trials.

## Contributions

MTA and SZD led the overall conceptualization of the manuscript and jointly drafted and refined the full narrative. Subsection-level conceptualization and writing were undertaken as follows: the section *Video-based Outcomes Recording for Ecological Validity* was conceptualized and written by MTA and SS; *AI/ML to Strengthen NRD Trial Endpoints* by GD; *Use of Electronic Devices for Ecological Momentary Assessments of Real-World Data* by MTA, JCD, SG, JAA and EAC; *Goal Attainment Scaling as a Personalized Endpoint* by GS, CJE, CC and KR; *Clinical Global Impressions Instruments* by MTA; *Reliable and Clinically Meaningful Change Values (RCI/MCID)* by KIB and PKT; *Multi-Domain Responder Index (MDRI)* by PKT; *Growth Scale Value Methodology* by EBK; *Natural-history Studies as Opportunity to Learn* by MTA; *Core Outcome Sets and COA Methodology* by MTA and CMO; *Parents' Expectations and Subjectivity* by FR, SG, JAA and JCD; and *Parents’ Perspectives on Trial Design and Outcomes* by SG and JAA. All other authors reviewed, critically revised, and approved the manuscript for intellectual content. Authors meet all four ICMJE authorship criteria, including substantial contributions to the conception or design of the work or the acquisition, analysis, or interpretation of data; drafting or critical revision of the manuscript; final approval of the version to be published; and agreement to be accountable for all aspects of the work.

## Declaration of generative AI and AI-assisted technologies in the writing process

During the preparation of this work, the authors used the Claude AI (Anthropic) Sonnet 4.6 model to assist with language editing and structural revision of sections. After using this tool, the authors reviewed and edited all content as needed and take full responsibility for the publication's content.

## Financial disclosures

Publication of this manuscript has been possible with the collaboration and funding of the 10.13039/501100007871European College of Neuropsychopharmacology (10.13039/501100007871ECNP). This research was supported [in part] by the Intramural Research Programme of the 10.13039/100000002National Institutes of Health (10.13039/100000002NIH). The contributions of the NIH author MTA are considered Works of the United States Government. The findings and conclusions presented in this paper are those of the author(s) and do not necessarily reflect the views of the NIH or the U.S. Department of Health and Human Services. CMO is supported by the 10.13039/501100004837Spanish Ministry of Science and Innovation, 10.13039/501100004587ISCIII (PI21/01929), 10.13039/100006301CIBER (CB/07/09/0023), the 10.13039/501100000780European Union (10.13039/501100008530ERDF Funds, NextGenerationEU), Madrid Regional Government, EU Structural Funds, the 10.13039/501100004963EU Seventh Framework Programme, the H2020 Programme under the 10.13039/501100010767Innovative Medicines Initiative 2 Joint Undertaking, 10.13039/100018693Horizon Europe (FAMILY, Psych-STRATA, Bootstrap), the 10.13039/100000025National Institute of Mental Health, Fundación Familia Alonso, and Fundación Alicia Koplowitz. The work of BC was supported by French government grants managed by the 10.13039/501100001665Agence Nationale de la Recherche under the France 2030 programme, reference ANR-22-EXPR0013 and ANR-23-IAHU-0010.

## Declaration of competing interest

The authors declare the following financial interests/personal relationships which may be considered as potential competing interests: SZD developed the EPICOG-SCH Brief Cognitive Battery in Schizophrenia and has provided consultancy services to the pharmaceutical industry (Sanofi-Genzyme and Jazz Pharmaceuticals, Merck Healthcare and Alexion, 2018–2025). CA was supported by the Spanish Ministry of Science and Innovation, Instituto de Salud Carlos III (ISCIII), co-financed by the European Union (ERDF Funds, NextGenerationEU), CIBERSAM, Madrid Regional Government, EU Structural Funds, the EU Seventh Framework Program, the EU H2020 Program under the Innovative Medicines Initiative 2 Joint Undertaking (PRISM-2, Grant No. 101034377; AIMS-2-TRIALS, Grant No. 777394), Horizon Europe, and the U.S. National Institute of Mental Health (Awards 1U01MH124639-01 and 5P50MH115846-03), as well as Fundación Familia Alonso and Fundación Alicia Koplowitz. CA has been a consultant to or has received honoraria or grants from Abbot, Acadia, Ambrosetti, Angelini, Biogen, BMS, Boehringer, Carnot, Gedeon Richter, Janssen Cilag, Lundbeck, Medscape, Menarini, Minerva, Otsuka, Pfizer, Roche, Rovi, Sage, Servier, Shire, Schering Plough, Sumitomo Dainippon Pharma, Sunovion, Takeda and Teva. JB is a full-time employee of Signant Health and may own company stock. GD is a shareholder and part-time employee of The Siesta Group. IDC has been a consultant to or has received honoraria from the European Research Executive Agency – European Union, Worldwide Clinical Trials and Clario, outside the submitted work. JCD has performed consultancy work for multiple pharmaceutical and biotechnology companies and declares no financial interest in their success or failure. EG declares no conflicts of interest. SHK declares no conflicts of interest. CMO has been a consultant to or has received honoraria from Angelini, BAP, Compass, Esteve, Exeltis, Janssen, Lundbeck, Neuraxpharm, Nuvelution, Otsuka, Pfizer, Rovi, Servier, Sunovion and Teva, outside the submitted work. SP has provided consultancy services since 2021 to Recordati, Biohaven Pharmaceuticals, Angelini Pharma, GW Research Limited, Innova Pharma, Swiss National Science Foundation (SNSF), Lundbeck, Otsuka Pharmaceutical, Agence Nationale de la Recherche (France), Aboca, Biogen, Menarini, Neopharmed Gentili, Beckley Psytech, Medpace, Acsel Health and the NIH (R21 Grant Co-PI, NCT03669315). CP declares no conflicts of interest. SKS has received speaker honoraria from Medice Arzneimittel Puetter GmbH & CoKG, Takeda and Janssen in the past 3 years, and has received funding as Co-PI from the GB-A Innovationsfond (UplusE and PeriPsych projects). DT declares no conflicts of interest. MV owns and operates Santium, an organization providing COA-related consulting services to the pharmaceutical industry. MKS has received research support from the U.S. National Institutes of Health, the Patient-Centered Outcomes Research Institute, the Brain and Behavior Foundation, Advanced Neuromodulation Systems, AbbVie Inc., and Alto Neuroscience. She serves on a data safety monitoring board for a study funded by the National Institute of Mental Health and, in the past 3 years, has consulted for or served on advisory boards for AbbVie Inc., Alkermes, Alto Neuroscience, Axsome, Boehringer-Ingelheim, Bristol Myers Squibb, Johnson & Johnson, Karuna Therapeutics, Neumora and Skyland Trail. She receives honoraria from the American Academy of Child and Adolescent Psychiatry and royalties from the American Psychiatric Association Publishing and Thrive Global. As scientific director of Fundación Síndrome de Dravet, SG has received grants or financial support from GW Pharma, Zogenix, Ovid Therapeutics, Encoded Therapeutics, Biocodex, Praxis, Stoke, Takeda, UCB, Epygenix, Jazz Pharmaceuticals and StrideBio for research or consulting; all honoraria were donated directly or indirectly to the Fundación Síndrome de Dravet. As president of the same foundation, JAA has received financial support from the same entities, with all honoraria donated directly or indirectly to the foundation. ASC is Co-Founder and CEO (pro bono) of the Women's Brain Project. EAC is an employee of Engrail Therapeutics, Inc. and may hold stock options. EBK has received funding from numerous commercial entities (full list available in manuscript) to consult on trial design or conduct clinical or laboratory validation studies in genetic neurodevelopmental or neurodegenerative disorders; all funds are directed to RUMC in support of rare-disease programs, with no personal remuneration and no relevant institutional financial interests. FR supports activities of the 22q11.2 patient organization “Wir sind 22Q″ and has received compensation for travel and lodging, and a talk fee from vitos Kliniken Hadamar. GS, KR and CC are employees of Ardea Outcomes, Inc. PKT is an employee of Ultragenyx Pharmaceutical, Inc. RL is an employee of Otsuka Pharmaceutical Development & Commercialization, Inc. SS is an employee of Origins, Ltd. CJE is a consultant to Ardea Outcomes, Boehringer Ingelheim, Clinical Outcomes Solutions, Cogstate, Lighthouse Pharma and Vanqua Bio. BC has received speaking fees from Janssen-Cilag, Eisai and Otsuka-Lundbeck, supervises a PhD student funded by a CIFRE fellowship (ANRT) in collaboration with Eurofins Biomnis. EB declares no conflicts of interest. SFM declares no conflicts of interest. IM declares no conflicts of interest.

## Appendix A Supplementary data

Supplementary Figure 1. Poster Presented at ECNP 2024. Multimedia component. 1

Supplementary Table 1. NRD_CT Solutions. Multimedia component. 2

Supplementary Table 2. Checklist of the challenges. Multimedia component. 3

## Footnotes

1 The National Institute for Health and Care Excellence (NICE) is an independent public body that provides national guidance and advice to improve health and social care in England, and other parts of the United Kingdom (UK). It develops recommendations on best practices, assesses new health technologies, and creates quality standards to ensure the UK National Healthcare System and social care system provides high-quality, cost-effective care.
