# Supplementary material for: Outcome strategies for clinical trials in Neuropaediatric rare diseases
Source: Neurosci Appl. 2026 Jul 10;5:107021. doi: 10.1016/j.nsa.2026.107021 (PMC13393641; doi:10.1016/j.nsa.2026.107021)
Supplement: Supplementary file 1 — Supplementary Figure 1. Poster Presented at ECNP 2024. Multimedia component. 1 [file mmc1.pdf]

37th ECNP Congress
21-24 September 2024
Milan, Italy

MILAN-P3412

OUTCOME RESEARCH STRATEGIES FOR CLINICAL TRIALS IN NEUROPEDIATRIC RARE DISEASES

Silvia Zaragoza Domingo (1)*, Kim I. Bishop (2), Inés del Cerro (3), Cristina Baeza (4), Marie-Liesse Bryche (3), Estíbaliz Arce Cirauqui (5), Maria T. Acosta, MD (6)

(1) Neuropsychological Research Organization, S.L. (Neuropsynchro), Barcelona, Catalonia, Spain, (2) Global Pharma Consultancy, LLC, PA, USA, (3) Department of Psychology, Medical School, Catholic University of Murcia (UCAM), Murcia, Spain, (4) CBA Formación Educational and Health Consulting, Barcelona, Spain, (5) Engrail Therapeutics, Inc., San Diego, CA, USA, (6) Undiagnosed Disease Program, National Human Genome Institute, National Institutes of Health, Bethesda, MD, USA

*** Corresponding author: szaragoza@psyncro.net**

BACKGROUND

Challenges for clinical trials (CTs) in neuropediatric rare diseases (NRDs) include difficult access to adequate study patient samples, phenotypic variability, and limited efficacy of conventional study designs. There are further challenges around patient's evaluation due to disease severity and the difficulty to evaluate efficacy in developmental state of the abilities under study.

The selection of clinical outcome assessments/measurements (COAs/COMs) in randomized CTs is usually an area of high interaction between several disciplines and other stakeholders. Furthermore, in rare disorders research, the inclusion of statistical experts, patient advocacy groups as well as translational scientists are of particular relevance considering the scarcity of data, impact on families, and lack of validated translational biomarkers to steadily progress towards the later clinical phases of drug development.

OBJECTIVES & METHODS

Our aim is to describe the current landscape for selecting COAs/COMs and to explore current/future methodologies in neuropediatric rare disease CT. We propose to: 1) identify the stakeholders dealing with COAs in CTs, 2) describe available sources to support COAs/COMs selection, 3) describe the most innovative strategies used for COAs/COMs selection, and 4) collect proposed innovative COAs/COMs developments for future study designs.

Papers were extracted from a targeted literature. We present a summary using a semi meta-narrative review system according to: 1) challenges in rare diseases, 2) proposed solutions, 3) stakeholders, 4) existing outcomes and selection strategies, 5) existing endpoints, and 6) innovative paths. All elements found were organized within a predefined framework. The literature search included all the pediatric rare diseases and core outcome set (COS) initiatives[2]. A search in biomedical public databases (PubMed, Google Scholar, Web of Knowledge, SCOPUS, etc.) was conducted using the following keywords: "clinical trials", "review", "methodology", "rare diseases", "orphan diseases", "pediatric", "paediatric", and "child". For search in CTs public databases (clinicaltrials.gov, COMET, etc.), the terms "rare diseases" or "orphan diseases" were used.

TABLE 1. RELEVANT SOURCES FOR COA INSTRUMENT IDENTIFICATION/SELECTION

STRATEGY	EXAMPLES	STRENGTH	WEAKNESS
**Critical**			
**RA Scientific Guidelines** *(See Table 2)*	• EMA<br>– Clinical investigation of medicinal products for the treatment of Duchenne and Becker muscular dystrophy<br>• FDA<br>– Clinical Investigations Involving Children<br>– Pediatric Rare Diseases--A Collaborative Approach for Drug Development Using Gaucher Disease as a Model; Draft Guidance for Industry<br>– Human Gene Therapy for Rare Diseases	• Clarification and directions for future research and regulation on innovative study designs<br>• Mentions need to validate new COAs<br>• Consideration of main principles in developing novel efficacy endpoints in gene therapy trials	• Scientific guidelines addressed main disorders but not specific diseases (i.e., epilepsy) or main topics of interest (ethics, specific therapies, etc.)
**Supportive**			
**Task Force with Different Relevant Stakeholders**<br>**International Consortia**	• Orphanet<br>Offers an inventory of rare diseases and directory of resources including ongoing research projects, CTs, registries, and biobanks	• Powerful resource and repository	• Not described in the literature
	• Collaborative Gaucher Disease NFAND scale<br>Scoring systems used to assess its severity in type 1 Gaucher disease (GD1)	• Standardizes assessment of learning difficulties<br>• Used as gold standard	• Too over-controlled<br>• Too simple
	• Cerliponase Alpha pivotal clinical trial in enzyme replacement therapy (NCT01907087 and NCT02485899).<br>First approved treatment using an independent group of historical controls.	• Resolving main methodological difficulties working with historical controls, allowing compare patient's absolute scores changes in four domains — motor skills, language, vision, and seizure — to matched historical controls	• Strong and efficient communication between stakeholders is required to ensure the application of innovative solutions
	• Children Tumor Foundation (CTF) – Improving Efficiency in Response Evaluation in Neurofibromatosis and Schwannomatosis (REiNS)	• Efforts to standardize clinical trial endpoints by supporting Response Evaluation in REiNS<br>• Organization of special meetings to engage patients in the design of CTs	• Not described in the literature
**Past Completed CTs Review**<br>**Systematic Reviews and Meta-analysis**	• Cochrane Library Plus from Cochrane Foundation<br>- Review of used methodology and COA in RCT.<br>- Systematic Reviews of Pharmacological Treatment in Hunter's Syndrome, Friederich's Ataxia, etc.	• Summary of the *State of the Art*<br>• High quality systematic reviews<br>• Strong methodological protocols to identify and analyze existing CTs.<br>• Comprehensive analysis of used outcomes including efficiency in research.	• Study selection bias selection criteria<br>• Not focused on COAs efficiency or sensitivity<br>• Focus on "Intervention" rather than methods<br>• Limitations of analyzing evidence restricted to prospective randomized CT, especially when dealing with chronic rare diseases
**Literature Review for Existing Core Outcomes Set (COS) Projects**<br>**Standards for outcomes selection**	• COMET Initiative<br>Database of projects on COS<br>• European College of Neuropsychopharmacology (ECNP) Outcomes Research in Neurosciences Group & TWG Outcomes Research at Early-trials in Neurosciences<br>Promoting the use of standard methods in COA selection in neurosciences CTs	• Database of Systematic Review Works<br>• Identification of Core Outcomes Set projects (different types) Pediatric Pain - edIMMPACT, OMERACT, PIA<br>• Complete and firsthand information can be obtained<br>• Support on application and innovation by author with continuous validation of Composite Scores	• Potential study selection bias<br>• Health technology assessment oriented<br>• Limited dissemination *(example of COS in Pain; Connelly et al, 2019)*<br>• Challenges on interpretation of outcomes/endpoints *(Seattle Children's presentation)*<br>• Copyright limitations<br>• Potential conflict of interests
**Instrument Libraries**<br>**Explore available Instruments**	• Rare Disease COA Resource (RD-COAR).<br>Resource by C-PATH aiming to simplify COA selection for use in RD	RD-COAR<br>• Information on the support of clinical endpoints for CTs design (current and historical)	• RD-COAR from C-PATH is the unique providing free summary information about the validity of the instruments compared to other repositories
	• Mapi Trust Foundation COA/eCOA Library (eProvide)<br>Online library specialized in COAs with over 5,111 results on NRD indications<br>• BiblioPRO<br>Repository of >2,400 instruments, that includes 45 COAs in NRD	Mapi Trust Foundation<br>• Facilitates access to information to all stakeholders in the field of COAs<br>• Useful to collect information of specific instruments<br>• Useful to know copyright holder<br>• Useful to know available linguistic validations<br>• Information regarding use of COA in approved labelling	• The information is not always updated<br>• Access requiring subscription<br>• Available translations might not correspond to the same COA version
	• SRLAB Rehabilitation Measures Database<br>- COA library, including full description of instruments<br>- 500+ measures supported by clinical experts and researchers available for clinical use	• Comprehensive repository of existing instruments regardless therapeutic area	• Not described in the literature
**Public CT Databases**<br>**Identification of COAs included in research protocols**	• US Registry and aggregate database analysis of output (AACT Database)	• List of past and ongoing studies including mention of endpoints and COAs<br>• AACT makes it easier to analyze data from multiple studies, including protocol and results	• Limited information about COAs other than descriptions<br>• No information about efficiency of the instruments<br>• AACT aggregate summaries are only available for a limited number of diseases
	• EU Registry<br>• WHO ICTRP Registry CT<br>• JPRN Japan Registry of CT	• Comprehensive public registry	• Not described in the literature
**Considering Ongoing Innovative Approaches**<br>**Item Banks**	• C-Path initiative (Health Measures)<br>A research resource infrastructure for use and interpretation of person-centered health outcomes (four measurement information systems, funded as separate NIH programs) for researchers and health professionals<br>Example: RD-COA Rare Disease Clinical Outcome Assessment Consortium *(see above on Instrument Libraries)*	• Development of novel COAs based on item banks using rash methodology (i.e., PROMIS)	• Not described in the literature
**Good Evaluation Practices**<br>**Outcomes Quality**	• ISPOR Guidance Task Force<br>- Clinical Outcomes Assessment Emerging Good Practices Task Force (2015)<br>- Clinical Outcome Assessments: Conceptual Foundation—Report of the ISPOR Clinical Outcomes Assessment – Emerging Good Practices for Outcomes Research Task Force (2017)<br>• COSMIN - Quality Checklist to Evaluate PROs<br>• EMPRO - Standard Assessment Checklist for PROs	• Useful to rate the quality of measurement instrument(s)<br>• Useful to guide selection when there are several options	• Evaluation tools are not for the specific use in the context of CTs
**Database of Research Projects Public Funded (past/current)**<br>**Collaborative Funded Projects**	• CORDIS Database for EU funded projects<br>• EU COST Action Funded Projects<br>Example: MINDDS Maximizing Impact of research in Neurodevelopmental Disorders	• Information about public funded projects in health research including innovative medicines initiatives (IMI)	• Development and validation of technology may need additional funding and partnership with industry stakeholders
**Specific projects on methodologies for CTs in small populations**	• EU FP7 Innovative Methodology for Small Populations Research (InSPiRe) project<br>• EU FP7 The Integrated Design and Analysis of Small Populations Group Trials (IDeAl) project in pediatric Oncology<br>• EU FP7 The Advances in Small Trials Design for Regulatory Innovation and Excellence (Asterix) project	• ASTERIX: Improved the use of patient level information and perspectives as Goal Attainment Scaling and better use of patient registries	• Not described in the literature

CDISC, Clinical Data Interchange Standards Consortium; CHMP, Committee for Medicinal Products for Human Use; COA, Clinical Outcome Assessments; C-Path, Critical Path Institute; CT, Clinical Trial, EMA, European Medicines Agency; ECNP, European College of Neuropsychopharmacology; FDA, Food and Drug Administration; PoC, Proof of Concept; PRO, Patient Reported Outcome; PCORI, Patient-Centered Outcomes Research Institute; PROMIS, Patient-Reported Outcomes Measurement Information System; RA, Regulatory Agencies; RWD, Real World Data

TABLE 3. MAIN GAPS AND CORRESPONDING INNOVATIVE STRATEGIES TO IMPROVE RESEARCH EFFICIENCY IN RARE PEDIATRIC CTs

MOST FREQUENTLY MENTIONED GAP / ISSUE	SUMMARY OF PROPOSED SOLUTIONS
**Extremely low patient numbers**<br>• *Limited access to study sample*<br>• *Problems recruiting concurrent controls (ethical concerns)*	• Enhancing statistical efficiency at the design phase, requiring fewer patients to conduct a robust evaluation (Tingley, 2018)<br>• Use of adaptive designs to minimize the sample size required to yield a result, yet maintain statistical validity (Gallo, 2006)<br>• Include sensitivity analysis to assess robustness of the results (Thabane et al., 2013; Ghadessi et al., 2020)<br>• Using Bayesian rather than frequentist analysis methods, allowing more statistical flexibility, to combine information (e.g., historical information with study information) (Tingley, 2018; Ghadessi et al., 2020)<br>• Replicate study results in different settings (van der Lee, 2008)<br>• Use of historical control data (RWD, medical charts, patient registries, natural history trials, already completed CTs) in life threatening diseases with no credible control arm or invasive therapies (Ghadessi et al., 2020, Vockley et al., 2023)<br>• Interaction with biostatisticians to guide and conduct appropriate designs and analysis (e.g., piloting exploratory studies prior to pivotal trial, conduct prospective, observational, non-interventional studies early in the clinical development program) (Videnovic et al., 2023)
**Incomplete understanding of disease pathology**	• Use of natural history data to better understand the progression (e.g. define cut-off points for CT enrollment, note potential limitations due to disease variability, changes in environmental factors and standards of care), to inform a specific context for drug use, efficient clinical trial design, refine parameter estimations, adapt the study to clinical condition, and streaming the pathway towards regulatory acceptance (Mulberg et al 2019)<br>• Engagement with specialist managing patients with the disorder, patient and caregiver advocacy groups, regulatory experts and agencies (Videnovic et al., 2023; Vockley et al., 2023)
**Existing barriers to advancing knowledge & treatments in rare diseases**	• Efforts to resolve methodological challenges (e.g., creating innovative methods to address minimal sample size requirements and maximizing the proportion of patients receiving active treatment) (Gagne et al, 2014); Registry-based randomized trials are an emerging design in pediatric rare disease settings<br>• Include regulatory bodies at the design stage to discuss and explore potential concerns, designs, previous regulatory decisions and reports (Ghadessi et al., 2020)
**Lack of established endpoints**<br>**Insufficient COS definitions**	• Barriers to efficient and effective CTs which can meet regulatory requirements for drug approval<br>• Consensus-based COS decisions including different stakeholders (Mulberg et al, 2019)<br>• Use of natural history data to inform about COAs selection in the context of event rate and variability of disease presentation to ensure sample size for sufficient statistical power to detect efficacy (Mulberg et al, 2019; Vockley et al., 2023)<br>• Use of clinical trial simulation to quantify and evaluate design operating characteristics and possible decisions, selecting methods for bias control and how to compare measurement between groups addressing interfering events (rescue medication, drop-outs, death, non-adherence, non-compliance, etc.) (Ghadessi et al., 2020)
**High phenotype variability & High degree of clinical variability among rare disease patients**	• Extremely different clinical manifestations in metabolic disorders with different responses to given interventions require more pragmatic approach on study designs (Tingley, 2018)<br>• More pragmatic approach to evaluate treatment effectiveness (e.g., pragmatic CTs, observational studies and hybrid designs incorporating randomization and systematic observation) (Tingley, 2018)<br>• Incorporate within-patient measurements, include crossover trial and N-of-1 trial (Tudur et al., 2014)

STAKEHOLDERS IN RARE DISEASES AND ACTIVITIES

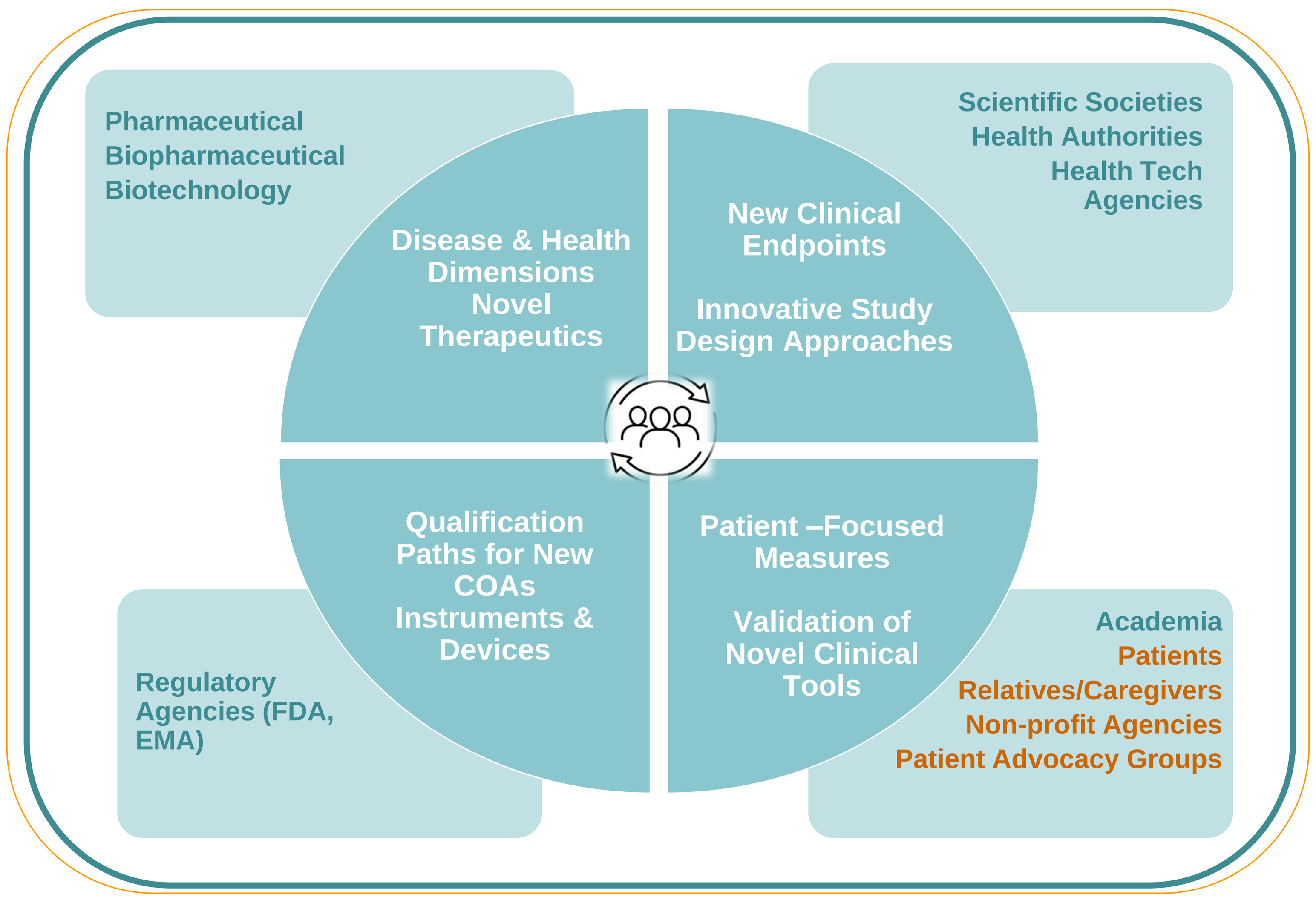

TABLE 2. RELEVANT STAKEHOLDERS IN NRD FIELD

STAKEHOLDER	ROLE	EXAMPLE
**Regulatory Agencies**	**Issuing Specific Regulatory Research Guidelines**	• **EMA**<br>- 2018: Extrapolation of efficacy and safety in paediatric medicine development - Scientific guideline<br>- 2017: Pediatric Gaucher disease: a strategic collaborative approach from EMA and FDA<br>- 2006: COMMITTEE FOR MEDICINAL PRODUCTS FOR HUMAN USE. Guideline on clinical trials in small populations<br>• **FDA**<br>- 2023: FDA Draft Guidance for Industry: Adjusting for Covariates in Randomized Clinical Trials for Drugs and Biologics with Continuous Outcomes<br>- 2019: Demonstrating Substantial Evidence of Effectiveness for Human Drug and Biological Products, Guidance for Industry, Draft guidance, US Department of Health and Human Services, Food and Drug Administration, Center for Biologics Evaluation and Research (CBER), Center for Drug Evaluation and Research (CDER)<br>- 2019: FDA Draft Guidance for Industry Rare Diseases: Natural History Studies for Drug Development<br>- 2019: FDA Draft Guidance for industry Rare Diseases: Common Issues in Drug Development<br>- 1983: The Orphan Drug Act (ODA) is a significant piece of legislation in the United States aimed at encouraging the development of drugs for rare diseases, often referred to as orphan diseases, providing incentives to pharmaceutical companies to develop orphan drugs
	**Promoting Innovation**	• **EMA**<br>- European Union Regulation (EC No 141/2000 for Orphan medicinal products (OMPs), providing incentives for research granting 10 (+2) years of marketing exclusivity among other financial and scientific provisions granted (Horgan, 2020)<br>• **FDA**<br>- CDER ORISE research project. Mitochondrial Symposium: to overcome significant challenges in designing and conducting adequate and well-controlled NRD trials, to support innovative trial designs and analyses, justified, and able to distinguish the effect of a drug from other influences (Morris, Lee, & Wang, 2019)
	**Guidance for development of COAs**	• 2014: FDA - FDA Patient-focused drug development guidance documents (PFDD)
	**Endpoint Qualification for CTs**	• FDA COA Qualification Program (updated biannually). Examples in NRD are:<br>- DDT-COA-000032, Abilities Captured through Interactive Video Evaluation (ACTIVE)-seated.<br>- DDT-COA-000103, ActiMyo®<br>• EMA: IRIS Platform - EMA's (CHMP) scientific advice to support the innovative development methods qualification<br>- Example: EMA Public available Qualification Report for New Endpoint in Duchenne Muscular Dystrophy studies
	**Repository of Endpoints Mentioned in Approved Labelling**	• FDA: Clinical Outcome Assessment (COA) Compendium - Approved Labelling COA Compendium - COAs already evaluated in the DDT COA Qualification Program or from NME labeling. COAs mentioned as PROs, ObsROs, for approved drugs in the Division Of Rare Diseases And Medical Genetics (DRDMG)<br>- Example: Mucopolysaccharidosis (different forms) Walking distance measured by a PerfO, 6 Minute Walk Test (6MWT) in the approval of Aldurazyme, Elaprase, Vimizim and Naglazyme
**Policy Makers**	**European Union**	• Setting measures to encourage pharmaceutical trials in children by legislation, which has been in effect since 2007 (van der Lee, 2008)
**Advocacy Groups (family/patients)**	**Variety of Roles**	• Different roles to support families, search CTs, facilities to patients, boost genetic research
**Associations & Scientific Organizations-Societies**	**Acceleration innovation in CTs and endpoints**	• The Human Genome Project<br>• Faster Cures<br>• Dravet Foundation-Research Grants to Develop or Improve Outcome Assessments in Patients<br>• Pediatric Rheumatology International Trials Organization (PRINTO)<br>• The Pediatric Rheumatology Collaborative Study Group (PRCSG)
	**ICH**	• International Conference on Harmonization Expert Working Group<br>- 2024: Clinical investigation of medicinal products in the pediatric population E11<br>- 2000: ICH Harmonized Tripartite Guideline: Choice of Control Group and Related Issues in Clinical Trials E10
	**Evaluation**	• Cincinnati Children's – Patient self-management indicators could be used as clinical endpoint<br>- Identification of domains on self- management: pain control, adherence, resources & support, multicomponent<br>• Vector GeneTherapy (PaVe-GT) platform:<br>- Tests whether gene therapy trial start-up efficiency could be improved, using a standard approach
	**Improve Outcomes Selection**	• Canadian Inherited Metabolic Diseases Research Network (CIMDRN Canadian Group)<br>- Pediatric multidisciplinary research network that generates evidence to improve outcomes and health care services for children with inherited metabolic diseases using large multi-centric cohort of children
	**Promote Patient Registries**	• Global TOPP Registry TOPP to track outcomes in specific diseases<br>• ECFSPR (European Cystic Fibrosis Society Patient Registry) a qualified for pharmacoepidemiology study would allow their use for regulatory purposes (Ollivier, 2017)<br>• The PARTNER project (Paediatric Rare Tumours Network - European Registry): very rare tumors children and adolescents
	**Boost investment**	• SIOP Europe- the Europe Society for Paediatric Oncology / European Society of Pediatric Oncology (ESPO) It is the only pan-European organization representing all professionals working in the field of childhood cancers influencing policies to increase incentives for the development of new treatments for rare diseases
	**Patient advocacy organization dedicated to the organizations**	• National Organization for Rare Diseases (NORD)<br>- Programs providing research grants (at least two FDA-approved therapies)<br>- Promoting the launch of disease-specific registries to support research<br>• EURODIS: Non-profit alliance of over 1000 rare disease patient organizations from 74 countries<br>- Creation of IRDiRC consortium *(see below)*<br>• Global Genes Non-profit advocacy organization dedicated to support individuals and families affected by rare and genetic diseases. Fundraiser campaigns.
**Consortia with Different Stakeholders (Academia / Foundations / Researchers / National Health Systems)**	**Consensus-based outcomes selection and validation**	• RD-COA Consortium at C-Path: Founded in 2022, enables precompetitive, multi-stakeholder collaboration aimed at identifying scientifically sound tools and methodologies for collecting clinically meaningful outcomes data in treatment trials for rare diseases<br>• Available in Pediatric Pain: PedIMMPACT (McGrath et al., 2008), OMERACT (rheumatology)<br>• Steps towards harmonization clinical development of medicines in pediatric ulcerative colitis (Sun, 2014)
	**Accelerate Knowledge Generation Improve Study Designs**	• IDeAL project consortia (EU Funded)<br>• TREAT-NMD – Excellence network in Neuromuscular Disorders<br>• ISCTM Orphan Diseases Working Group in CNS (Chairs: J. Bosner and G. Pandina). Five publications summarizing the *state-of-the-art* in NRD CTs
	**Demonstrating Effective Use of Historical Data**	• Example: Development of Cerliponase Alfa Enzyme Replacement Therapy (ERT) for the treatment of Ceroid Lipofuscinosis neuronal type 2 (CLN2)<br>Use of historical controls to avoid individual differences interfering comparability of treatment groups with historical controls, adjustment for co-variables, adaptation of the analysis to demonstrate a significant effect of the intervention (Mulberg, 2019; Shulz, et al, 2018)
	**Identify gaps, produce guidelines, recommendations and resources**	• The International Rare Diseases Research Consortium (IRDiRC)<br>• Bespoke Gene Therapy Consortium<br>• The International Society for CNS Clinical Trials and Methodology (ISCTM) –Orphan Disease Working Group (also in *Accelerate knowledge generation and improve study designs*)
**Pharma Industry & Biotech Companies**	**Promote regulatory environment**	• PHARMIG Association of Austrian Pharmaceutical Industry: Submission of feedback on the Roadmap for the Evaluation of the legislation on medicines for children and rare diseases (medicines for special populations). 8 January 2018. (Horgan et al., 2020)
**Technological Partners**	**Digital Health Innovation**	• Accelerate Electronic Devices as Clinical Care tools and Endpoints for CT

EMA, European Medicines Agency; C-Path, Critical Path Institute; COA, Clinical Outcome Assessments; CHMP ,Committee for Medicinal Products for Human Use; CDISC, Clinical Data Interchange Standards Consortium; ObsROs, Observer-reported outcomes; PerfO, Performance Outcome; PRO, Patient Reported Outcome; ClinROs, Clinician's Reported Outcome; RWD, Real World Data; PCORI, Patient-Centered Outcomes Research Institute; PROMIS, Patient-Reported Outcomes Measurement Information System.

TAKE-HOME MESSAGES

- **Pediatric RD is a field with a high level of interaction among stakeholders where patient-advocacy have a special driving role.,**
- **This review identified +28 different gaps in NRD COAs research limiting the progress in the advance of novel therapeutics ,**
- **Innovative solutions for COAs can be transversal and applied across different RD therapeutic areas showing similar research needs,**
- **Pre-competitive efforts are relevant to accelerate innovation and advancement of COAs development,**
- **Sustainability of the innovation i.e. investment either public or private is needed to progress in the methodological solutions,**
- **It is useful to summarize and evaluate existing resources for NRD in order to identify synergies and reduce duplicative efforts,**
- **Given the reduced number of patients, future solutions are needed to speed up the validation/qualification of novel COAs as collaborative efforts.**

KEY REFERENCES (A full list of papers included in the review can be requested to the corresponding author).

Ghadessi, M., Tang, R., Zhou, J., Liu, R., Wang, C., Toyoizumi, K., Mei, C., Zhang, L., Deng, C. Q., & Beckman, R. A. (2020). A roadmap to using historical controls in clinical trials – by Drug Information Association Adaptive Design Scientific Working Group (DIA-ADSWG). *Orphanet Journal of Rare Diseases, 15*(1), 69. doi: 10.1186/s13023-020-1332-x
Mulberg, A. E., Bucci-Rechtweg, C., Giuliano, J., Jacoby, D., Johnson, F. K., Liu, Q., Marsden, D., McGoohan, S., Nelson, R., Patel, N., Romero, K., Sinha, V., Sitaraman, S., Spaltro, J., & Kessler, V. (2019). Regulatory strategies for rare diseases under current global regulatory statutes: a discussion with stakeholders. *Orphanet Journal of Rare Diseases,14*(1), 36. doi: 10.1186/s13023-019-1017-5
Thabane, L., Mbuagbaw, L., Zhang, S., Samaan, Z., Marcucci, M., Ye, C., Thabane, M., Giangregorio, L., Dennis, B., Kosa, D., Debono, V. B., Dillenburg, R., Fruci, V., Bawor, M., Lee, J., Wells, G., & Goldsmith, C. H. (2013). A tutorial on sensitivity analyses in clinical trials: the what, why, when and how. *BMC Medical Research Methodology, 13*:92. doi: 10.1186/1471-2288-13-92.
Tingley, K., Coyle, D.,Graham, I. D., Sikora, L., Chakraborty, P., Wilson, K., Mitchell, J. J., Stockler-Ipsiroglu, S., Potter, B. K., & Canadian Inherited Metabolic Diseases Research Network. (2018). Using a meta-narrative literature review and focus groups with key stakeholders to identify perceived challenges and solutions for generating robust evidence on the effectiveness of treatments for rare diseases. *Orphanet Journal of Rare Diseases,13*(1), 104. doi: 10.1186/s13023-018-0851-1.
Tudur Smith, C., Williamson, P. R., & Williamson, P. R. (2014). Methodology of clinical trials for rare diseases. *Best Practice & Research Clinical Rheumatology, 28*(2), 247-262. doi: 10.1016/j.berh.2014.03.004
van der Lee, J. H., Wesseling, J., Tanck, M. W. T., & Offringa, M. (2008). Efficient ways exist to obtain the optimal sample size in clinical trials in rare diseases. *Journal of Clinical Epidemiology, 61*(4), 324-330. doi: 10.1016/j.jclinepi.2007.07.008
Videnovic, A., Pfeiffer, H. C. V., Tylki-Szymanska, A., Berry-Kravis, E., Ezgü, F., Ganju, J., Jurecka, A., & Lang, A. E. (2023). Study design challenges and strategies in clinical trials for rare diseases: lessons learned from pantothenate kinase-associated neurodegeneration. *Frontiers in Neurology, 14*:1098454. doi: 10.3389/fneur.2023.1098454
Vockley, J., Burton, B., Jurecka, A., Ganju, J., Leiro, B., Zori, R., & Longo, N. (2023). Challenges and strategies for clinical trials in propionic and methylmalonic acidemias. *Molecular Genetics and Metabolism, 139*(3), 107612. doi: 10.1016/j.ymgme.2023.107612

DISCLOSURE

Silvia Zaragoza Domingo, PhD is a research consultant and recently worked for WCG Clinical, Sanofi R&D and Jazz Pharmaceuticals. Inés del Cerro served as a consultant for Worldwide Clinical Trials. Kim I. Bishop, Ph.D. recently served as a consultant for Zogenix, Medtronic, UCB Pharma, Stoke Therapeutics.
