# Supplementary material for: Outcome strategies for clinical trials in Neuropaediatric rare diseases
Source: Neurosci Appl. 2026 Jul 10;5:107021. doi: 10.1016/j.nsa.2026.107021 (PMC13393641; doi:10.1016/j.nsa.2026.107021)
Supplement: Supplementary file 2 — Supplementary Table 1. NRD_CT Solutions. Multimedia component. 2 [file mmc2.docx]

**Supplementary Table 1.** Raw table of brainstormed outcome strategies piloted for Neuropediatric Rare Diseases in clinical contexts.

| **STRATEGY**  **(problem)** | **DESCRIPTION** | **EXPERIENCE & CONS** |
| --- | --- | --- |
| 1. Videorecording of participants performing pre-defined instrumental activities. | Clinicians design an activity for patients (PerfO) standardizing the process as much as possible.  Activities are adapted to patient baseline skills and within the normal home routine (e.g., adding cereals in a bowl in an increasing demand according to the patient’s age, walking capacity with different walking aids, dressing themselves, brushing their teeth, etc.).  Parents administer the activity at home and record video while performing. Clinicians provide a script to parents to guide the activity including recording instructions.  The periodicity of recording is set, for instance, recording the patient performing the “activity” every three months. Parents/Caregiver can record several times the patient doing the activity and select and send in the best performance video. | Positive experience during the ecological value (showing how behavior is affected in real-life or with real-life tasks).  As a complement to more standard measures such as Bayley Scales of Infant and Toddler Development (Bayley, 2014) or other PerfOs used in clinical settings.  Videos that parents obtain in the patient’s everyday life are much better than the observations obtained from a neuro exam at the clinic.  Ethological methodology can be used to analyze/categorize and score the patient’s behavior recorded by video. There are some experiences already in clinical research. Video recording to measure outcomes was applied in past clinical trials as in Tourette Syndrome for tics severity (NCT02679079) and in Muscular Atrophy Type 1 for motor independence (NCT03461289; National Institutes of Health 2021, and 2022).  Available software free of use BORIS (Behavioral Observation Research Interactive Software) that lows a user-specific coding environment to be set for a computer-based review of previously recorded videos or live observations and share among observers/collaborators (Friard & Gamba, 2016, 2016).  Video recording can compensate for floor/ceiling effects of standard tests.  **CONS**  It is difficult to standardize the instructions and to harmonize among patients.  Further harmonization of the process is needed.  Depending on the requirements, this can be a challenge for the available patients in subpopulations with certain concomitant impairments. Additional selection criteria limiting even more participation.  Videorecording can be expensive. Video brings the proof that the behaviour is conducted and at what level. Video recordings can be expensive if a monitoring device is needed (e.g. camera for sleep monitoring), however nowadays mobile phones are accessible to everyone at different costs.  Proposing specific instrumental activities might result in a number of errors.  Con: Return on Investment for recordings are not easily understood. Implementation of recordings need to be harmonized across sites, patients/parents, which can add to equipment costs.  Con: Regulatory agencies need to understand what to do with the recordings once obtained. If nothing, then centralized rating can justify the benefit of recordings. |
| 1. AI & Machine Learning Approach | Parents can collect data and one can analyse it as *big data*.  Several recordings of the same activity and choosing the best one to present as study endpoint.  AI initiatives and collection of video data for validation purposes would require a large amount of data. Other groups are working on similar ideas. | Consider exploring this to model the slope of evolution of the disease in standards of care. Can be useful to describe patient trajectories under different treatments.  Can be interesting to build a collaborative project with the aim to collect larger samples and data. Having trajectories of small number of patients can be an option for AI/ML?  Collect data at international level in a central database (e.g., in ADHD this is being conducted).  Limited number of available patients with the disease that can be mitigated by collecting data from a range of patients from normal to low performance due to other diseases.  AI offers different options, and all require a large amount of data. Models like ChatGPT are not applicable, as those models are based on language.  Aggregating case-information: Recording and collecting information along a patient's life is very interesting as the doctors keep track of patients since childhood to adulthood. Many of the patients die eventually but doctors keep records of information, interesting stablishing standards for natural history. How to reuse this information to have AI. Also, many of these patients have siblings which allows them to add more family members with the same mutation along the years.  **CONS**  Build models based in one patient will have a problem of general validity as the models derived will not be suitable for other patients.  An approach oriented to collect longitudinal data from patients, across development can bring information about development slope in several dimensions can be of interest. The system can learn as more patients are included in the database in a similar way as it occurs in clinical practice. Clinicians in this context can classify the slope as stable, deteriorating or improving.  Nowadays, endpoints derived from IA can be the case to be almost impossible to interpret the endpoint and require huge numbers to properly validate; often larger than is available in many of the validation studies and this is made worse in rare diseases where the number of patients is small.  Con: not enough data from rare disease patients to implement AI or the “big data” approach. |
| 1. Continuous momentary recording of patient’s functionality | Acquisition of continuous data through devices as wearables. | Limited experience.  **CONS**  Difficulty of patients with disability or behavioural problems to wear devices during long periods.  Acquisition of a lot of information/data and not clear definition about how to use it as clinical endpoint in specific diseases. |
| 1. Use of IPADs, and APPs to collect continuous information | Parents using IPADs to show to the patient’s performance at different time points.  Software inserted into the smartphones can also prompt quick questions (PROs) about patient’s condition in a timely or random manner. | A group in Stanford is recording face recognition in Autism being used in current research supported in hand-handled devices as an tablets and smart watches. There are recordings of facial expression at specific time points along the day.  Can be a challenge for the available patients in some rare disease subpopulations.  Random recording during the day using specific software e.g., Q1.6 Software (Q1.6, n.d.).  FDA is specifically interested in patient reported outcomes in real time. Unsure if EMA is as keen at PRO data as FDA.  **CONS**  In some types of patient populations, it is difficult to implement tools to monitor specific outcomes.  Con: if developed from scratch it will take significant time and money to develop a data collection tool that is accepted by FDA/EMA |
| 1. Family-related agreement on outcomes in a more personalized approach instead of a group change. | Some patients' symptomatology might be shown as stabilized while others experience deterioration.  Outcomes discussed and aligned with the relatives living with the patients, establishing reasonable outcomes. For example, capacity to “stand up “, patient able to still eat his/her food.  Families can observe many of the benefits not captured with standard outcomes used in clinical trials (functionality measured in scales is quite limited and not always what family’s needs, need to discuss what is meaningful as an outcome.  Looking at group mean change can dilute the benefits observed in the patients who showed benefit.  No improvement in one domain (e.g., walking) can be discrepant to benefits observed in other domains.  Maintaining skills (not getting worse) can be seen as benefits in patients who theoretically will deteriorate due to the disease. | Goal attainment scaling (GAS) can be a method to apply in this field, aimed to personalize endpoints to each patient's possibilities. Personalized outcomes approach.  Focus on family needs, including endpoints and measurements of instrumental behaviours functionality in daily life (school, sports, leisure time, etc..).  More patient-family-centred outcomes regarding functional measures (how to define functionality in global sense and how this can be implemented).  **CONS**  Precision methods may not be accepted by regulatory authorities because every patient is measured differently and regulators do not have experience with this. |
| 1. Parents’ subjectivity and expectations for improvement. | Coping with the relative expectations of improvement to mitigate placebo effect.  Expectancies regarding active treatment effect is also an issue during the evaluation. Parents sub or over-estimation of the evolution is common. This affects substantially ObsROs used in the trials. | The clinical site developed explanatory materials for parents prior to trial start. For example, people feel that a gene therapy is going to act fast and if it does not happen then there is disappointment that can mask real improvements.  Caregiver’s Global Impression of Severity / Change (CgGI-S/C) necessary as an outcome in all protocols. This can be used as anchor to the new outcomes.  A specific scale of Caregivers expectancies (CgExp) could be included as well, as a measure at Baseline of the hopes of parents participating in the trials. This can be a measure to co-variate with the endpoints measured by CG, to control for over or under-estimation.  Parent input, much like caregiver input, would be beneficial to payers (USA). |
| 1. Alignment/agreement on outcome domains across different stakeholders, clinicians and parents | Clinicians and parents often have different domains in mind when considering the outcome. This is cause of confusion and misleading about treatment efficacy.  Parents can be more interested on functionality in real context (at school for instance) and clinicians more interested in reduction of signs and symptoms. | When possible, promote the consensus on outcomes for a given condition, using the standard methodology of Core Outcomes Set.  Once agreed in the outcomes, consensus can be also reached on the measurement methods.  **CONS**  Parents and clinicians are often at odds about desired goals. Parents may be at disadvantage with respect to understanding the mechanism of action of the studied drug and the effects it may realistically achieve.  Functional scales are being developed in some of the rare diseases but not all. Validation of instruments is important. |
| 1. Maturity Index to use as reference for body development. | A physiological index that can be used as objective evolution of the patient, regardless of the disease.  This could be used as co-variant of other clinical outcomes or to include in the endpoint algorithm. | Would need to be based on brain imaging or any routine measure of child evolution.  Option to use the quantitative EEG, which have normative data, but the problem is the brain disease itself affects EEG measures i.e. seizures.  **CONS**  Psychological scales can be used but the floor effect will be a limitation because it is based on the normative population without the disease. |
| 1. Natural History records. | Natural history databases can bring information about the natural evolution of the patients to be used as historical controls.  The issue is how we can capitalize this information in order to extract information to be used to take decisions in future research.  Introduce the trajectory of the patient along the disease. | Retrospective CGI of Severity/Change (R-CGI-S/C) to measure disease severity and change based on medical records. The R-CGI can be used to rate historical patients for reference.  However, nowadays requires validation and shows a high subjectivity (*validation works in course by MT Acosta at NIH, ahead of Publishing).*  Currently data extracted from medical history is used as a placebo arm in clinical trials of novel therapies. We can describe how at SAP level. |
| 1. Composite Measures | Multi Domain Responder Index (Tandon & Kakkis, 2021)  The multi-domain responder index: a novel analysis tool to capture a broader assessment of clinical benefit in heterogeneous complex rare diseases. | Important issues around composite endpoints as primary endpoints for demonstrating the efficacy of new drugs in clinical trials have been extensively discussed elsewhere (Bakal et al., 2015; Chi, 2005; Sankoh et al., 2014) with different situations according to the level of correlation among the different domains.  Experience: Composite scores need regulatory input and agreement LONG in advance prior to implementing clinical trials. Secondary endpoints should be the individual domains.  **CONS**  Composite score used can dilute changes in single domains. |
|  | Growth Scale Values (GSV) included at the Vineland-3 and BAYLEY Scales (Bayley, 2014; Sparrow et al., 2016)  Random items are selected from subscales, But that items might be not the most important for the patient at the time when the selection is done.  How this can be improved. Interesting to know more about how these values are better between this GVS and row scores. How this can become novel COAs. | Based on IRT methodology providing a clear advantage to the traditional, standardization based on normative samples to calculate the global score.  **CONS**  Methodology can vary according to heterogeneity of patients.  Needed to understand more about the advantages in clinical trials of using GSV changes in front of changes on raw scores. More education programs are needed to understand the advantages in front other tools.  Value of Vineland and BAYLEY as endpoint when the ceiling values are reached.  There is a bit of more clarity on how all these novel outcomes work compared with what we have. |
| 1. Validation of Global Impression Instruments in different rare diseases | CGI-S/C, and other alternative forms as CgGI-I/C or PGI-S/C and retrospective (R-CGI-S/C) require validation before its use in clinical trials.  Multidimensional Impression of Change instruments can be validated including a Global assessment as a dimension. | There are many experiences of developing Global Impression Instruments which include a Global impression together with specific dimensions covering specific symptoms for the condition.  This can be tailored for each disease and shared across researchers to validate and future use. |
| 1. Establish a Reliable and Clinically Meaningful Change value | Present individual values if the group is heterogeneous, and difficult to establish.  This parameter is needed for study design purposes in order to establish values like magnitude of effect of the intervention and use it in future trials. | Present individual values if the group is heterogeneous (e.g., of those who showed reliable and clinically meaningful improvement look at corresponding seizure frequency).  Standardize the process for calculating a Reliable Change Index (RCI) and Clinically Meaningful Worsening (CMW) in rare diseases.  Reliable change indexes (RCIs) and clinically meaningful worsening >80% and clinically meaningful improvement >90 and >95% were calculated in post hoc exploratory “hypothesis generating” studies for the BRIEF caregiver family of instruments used in Dravet syndrome and Lennox Gastaut syndrome (Bishop et al., 2021, 2023).  **CONS**  Based on normative population, and results can depend on the statistic methodology applied to calculate reference values for clinical change. |
| 13. Core Outcomes Set (COS) for standard clinical practice useful to clinician’s regular work. | Outcomes more adapted to clinical practices and standards of care and rehabilitation.  Find a novel method that merges information of trajectories when there are comorbidities and rehabilitation programs combined with treatments (probably existing similar in different countries). | This is the typical situation that is seen in clinical practice. Develop a follow up method considering comorbidities, measure of the impact of different treatments similar in different areas or even rehab practices. This would improve the natural outcomes that we have as ecological momentary assessments. There is a need for agreement on the outcomes to be used in clinical practice.  Families and other stakeholders can be involved on the decision on which outcomes to select (functional might be preferred or educational achievements, etc..).  All this data is collected constantly in a routine practice until they get very old, making these records very useful as a landmark. The management when they go older is not so different from the childhood. Centres collect all this information. |
| 14. Establish the Core Outcomes Set (COS) for specific diseases where already exists a common practice of using the same COAs. | In several NRDs, there exist a common practice to use specific instruments, although not explicit in any publication. Experts on the field could develop a full COS to document the process and tune the practice for future clinicians and researchers. | Researchers of Phelan McDermid Syndrome have a trend to use similar instruments, and other instruments are being developed. However, there is a lack of deep analysis of its gaps or future avenues to improve the tools.  This lack of background work prevents its use at regulatory level as there are FFP after years with little documented properties or efficiencies in clinical trials.  7-Step process may need to be adapted or versioned for NRDs. |
| 15. Increasing connexion with basic research | Establish a more fluent communication between basic researchers and clinical researchers for genetic research for instance. | Many of advances in clinical field can be attempted to transferred quickly to animal research by basic researchers. |
| 16. Engagement of Regulatory Agencies on the innovations | In specific conditions agreements with regulatory agencies can be feasible. | A number of areas can be mentioned with participation of Patient Advocacy Groups and FDA.  Mention case examples.  Composite endpoints, digital biomarkers, biomarkers, PROs/caregiver reported outcomes are examples  Experience (JC Diaz): for rare disease, especially paediatric diseases, it is imperative, time consuming and costly but of utmost importance to get regulatory agencies buy-in and acceptance on PROs, recordings of patient experiences, COMs, etc. in rare disease. Pharma must take ample time to develop and/or convince regulatory bodies that the proposed study endpoint is acceptable to measure the disease and determine efficacy |
| 17. Outcomes needed for testing novel treatments. | Case example Neuromodulation. Different types of neuromodulations (4-5) but there are not clear instruments to be used.  Collecting clinical data that can be used in the future. | CGI important assessment and then depending on the diagnosis.  Sometimes they neurofeedback as an outcome measure to monitor the patient with OCD problems following treatment. Developing a specific program of testing TOC spectrum. [The global assessment of OCD - PubMed](https://pubmed.ncbi.nlm.nih.gov/36007341/) (Pampaloni, 2022). |
| 18. Core outcome sets (COS) needed in complex diseases that have psychiatric and also inflammatory components. | Need for multi-level approach of measurement needed to get the full picture (including patient, neuropsychology, parents, biomarkers, etc..).  For a large percentage of these patients with OCD PANDAS and Autism there is a complex need for evaluation, simplicity and complexity otherwise there is not a sense of what the patients suffer. | CGIs always included for follow up. Also, for Autism, Phelan McDermid Syndrome and Paediatric Acute-onset Neuropsychiatric Syndrome (PANS) and Paediatric Autoimmune Neuropsychiatric Disorders Associated with Streptococcal Infections (PANDAS) are conditions that are characterized by a sudden and severe onset of obsessive-compulsive disorder (OCD), use instruments as CATs and ASD parents stress index and Montefiore Scary instrument), but also instruments for parents (a publication with all instruments). These conditions also include assessment of neuropsychology and neuro-inflammatory.  Need for multi-level assessment, NPS, clinical, biomarkers, etc.  Global assessment on OCD in comprehensive psychiatry. Need to standardize for comparability. |
| 19. Collect parents’ perspective on clinical trials design and outcomes. | Considering patient’s relative experience with clinical trials  Need to better understand patients/relative’s perspective when participating in clinical trials, and get ideas for risk mitigation in future designs,  Exit Interviews to tutors/relatives to know more about clinical trial experience.  Engage expert tutors/relatives as stakeholders in clinical trials design. | Relatives and tutors can provide important input to the design of clinical trials, by sharing what they experienced during the participation.  The method of Exit Interviews following a clinical trial, is a potential methodology to be implemented. This information can also be collected post-hoc from study participants.  A professional patient/tutor can be defined as someone that has participated in clinical trials (lived experience on measurement of health) or that has a scientific background and knowledge about the disease.  Patient Advocacy Groups can also have a relevant role on this activity by increasing the communication between them and pharma R&D.  CONS  Lack of scientific knowledge can be an issue, and for that reason it is interesting to consider involving experts on scientific topics around clinical trials design with experience on NRD in order to facilitate the innovation in the field. |
| 20. Standard COMs for the evaluation of rare diseases that have available treatment. | There is a need to have a set of Core Outcome Measures ready to evaluate patients that start treatment of rare diseases with available treatment. | During clinical practice there are cases that are diagnosed and start treatment, this brings unique opportunity to evaluate patients before and after treatment in a comprehensive way, learning how these patients improve once the treatment starts.  It will be interesting to start a COS project to decide and standardize in a consensus-based method the domains and instruments to use in this context. Useful across different neurodevelopmental disorders.  NIH-Toolbox for cognition can be an option to start? |
| *Abbreviations: ObsRO (Observer Reported Outcomes), PerfO (Performance-based Outcomes), AI/ML (Artificial Intelligence/Machine Learning), ADHD (Attention Deficit Hyperactivity Disorder), FDA (United States Food and Drug Administration), Neuropediatric Rare Diseases (NRD), NIH (National Institute of Health), COS (Core Outcome Sets), COM (Core Outcome Measures), R&D (Research and Development), OCD (Obsessive Compulsive Disorder), RCI (Reliable Change Index), CMW (Clinically Meaningful Worsening), CGI (Clinical Global Impression).* | | |
