# Supplementary material for: Outcome strategies for clinical trials in Neuropaediatric rare diseases
Source: Neurosci Appl. 2026 Jul 10;5:107021. doi: 10.1016/j.nsa.2026.107021 (PMC13393641; doi:10.1016/j.nsa.2026.107021)
Supplement: Supplementary file 3 — Supplementary Table 2. Checklist of the challenges. Multimedia component. 3 [file mmc3.docx]

**Supplementary Table 2.** Checklist of Limitations Acknowledged in the Context of Clinical Trials in Neuropediatric Rare Diseases (version 1.0). Adapted from: Acosta M.T, Acosta, M.T., Zaragoza Domingo, S., Arango, C. et al. ‘Methodological challenges in outcomes research for early-trials for implementation of new therapies in neuropediatric rare diseases.’ Orphanet J Rare Dis 20, 521 (2025). https://doi.org/10.1186/s13023-025-03814-0:

|  | **RECOGNIZED METHODOLOGICAL CHALLENGES** | **Yes/No** | **Solution** |
| --- | --- | --- | --- |
| 1 | Reliability of clinical and biological outcomes |  |  |
| 2 | Standardized testing, psychometric issues (e.g., floor effects) |  |  |
| 3 | Comparability of profiles of rare diseases subpopulations to neurotypical groups |  |  |
| 4 | Concerns of traditional neurological examination not sensitive enough to detect changes in clinical trials |  |  |
| 5 | High carer expectations of the new treatments (effect and time) |  |  |
| 6 | Patients/carers traveling long distances to the study sites |  |  |
| 7 | Variability across clinical presentations of the same conditions or at different disease stages |  |  |
| 8 | Different age ranges of the participants in the same study |  |  |
| 9 | Length of clinical trials are to be too short to see improvements |  |  |
| 10 | Interview-based assessments exploring adaptive behavior assessed on subjective evaluations by observers of patient’s skills, not based on performance evaluations. It is not possible to prove it with objective data (e.g., Vineland Adaptive Behavior Scales-VABS) |  |  |
| 11 | Comorbidities and treatment for comorbidities are confusing factors (e.g. treatment as rehabilitation programs) |  |  |
| 12 | Lack of definition of functionality in the context of rare diseases applicable in daily life |  |  |
| 13 | Outcomes based on Clinical Global Impressions (CGIs) unidimensional too simplistic without split in domains |  |  |
| 14 | Variable acceptance of innovative outcomes by regulatory agencies (EMA, FDA) |  |  |
| 15 | For the condition there not exist stablished clinical outcome assessment tools suitable for clinical trials (fit-for-purpose) or consensus-based core outcomes sets (COS) |  |  |
| 16 | Do natural history studies or cohort studies exist for this condition? |  |  |
| 17 | Other |  |  |
